# Influence of fruit maturity and variety on seasonal postharvest fungal diseases and vascular streaking in California Avocados

**DOI:** 10.1371/journal.pone.0351752

**Published:** 2026-06-25

**Authors:** Boris X. Camiletti, Claudia M. Asensio, Thiago A. Carraro, Giorgio Gusella, Eric D. Focht, Roman Pandey, Themis J. Michailides, Mary Lu Arpaia

**Affiliations:** 1 Department of Crop Sciences, University of Illinois Urbana-Champaign, Urbana, Illinois, United States of America; 2 Department of Botany & Plant Sciences, University of California Riverside, Kearney Agricultural Research and Extension Center, Parlier, California, United States of America; 3 Department of Plant Pathology, University of California Davis, Kearney Agricultural Research and Extension Center, Parlier, California, United States of America; 4 Department of Agriculture, Food and Environment (Di3A), University of Catania, Via S. Sofia 100, Catania, Italy; University of Duhok, IRAQ

## Abstract

This study evaluated the avocado varieties ‘Hass’, ‘GEM’, ‘Luna UCR’, ‘Eugenin’, ‘Flavia’, I27EG, I55B, I49LB, I54J, and I73WL to determine seasonal dry matter (DM) patterns and assess how harvest timing, storage duration, and variety influenced stem-end rot, body rot, vascular streaking, and fruit susceptibility. Fungal pathogens associated with postharvest decay were identified using morphological characterization and multilocus phylogenetic analyses based on ITS, tub2, tef1-α, GAPDH, and MAT1–2 regions. Four *Neofusicoccum* species (*N. australe*, *N. luteum*, *N. nonquaesitum*, and *N. parvum*), one *Colletotrichum* species (*C. perseae*), and two Diaporthe species (*D. baccae* and *D. foeniculina*) were identified from decayed avocado fruits. Susceptibility tests confirmed that *N. nonquaesitum* was the most aggressive pathogen on avocado fruit, followed by *C. perseae* and *D. foeniculina*. The diversity of pathogens causing postharvest rot highlighted the complexity involved in managing stem-end rot and body rot in avocado fruits. Seasonal patterns of dry matter (DM) accumulation were identified for new varieties, aiding in establishing these standards. The study explored the seasonal variation in DM percentages across different avocado varieties and their correlation with disease severity. Results showed that this parameter can help prevent fungal disorders in some varieties. This finding emphasized the importance of adhering to both minimum and potentially maximum maturity standards to minimize fruit diseases and disorders and optimize postharvest performance. Additionally, the seasonality of stem-end and body rots in California avocados appears to be more associated with the inherent susceptibility of the fruits at harvest time than with environmental factors. Overall, general seasonal patterns were difficult to determine, as final postharvest disease intensity depended on the variety and the disease.

## Introduction

Avocado (*Persea americana* L.) is a traditional crop in California with more than 21,000 ha mainly distributed in Ventura, San Diego, Santa Barbara, San Luis Obispo, and Riverside counties (California Avocado Commission 2021). California produced 138,500 tons of avocados in 2022/23, equivalent to a value of 487 million USD [1]. Several varieties of avocados are grown in California including ‘Hass,’ ‘Lamb Hass,’ ‘Bacon,’ ‘Zutano,’ ‘Fuerte,’ ‘Pinkerton’ and ‘GEM’. However, ‘Hass’ is the predominant one, accounting for approximately 95% of the total production. In fact, the other cultivars are grown to extend the harvest season: ‘Bacon’ and ‘Zutano’ are harvested in the winter, while ‘Lamb Hass,’ ‘GEM’, and ‘Pinkerton’ are harvested in the summer. ‘Hass’ avocados fill in the gap between these seasons [2]. The prevalence of the ‘Hass’ variety in the California avocado industry offers numerous advantages, including a long harvest season, superior eating quality, and consistent postharvest characteristics [2,3]. Nonetheless, it encounters several obstacles, including large tree size, low production efficiency, alternate bearing, diminishing fruit size as trees mature, susceptibility to harsh weather conditions, vulnerability to specific pests, and seasonal production that limits annual productivity [4–9]. The introduction of higher-yielding varieties could stabilize production, ensuring a continuous year-round avocado supply with varied nutritional and sensory qualities, while also reducing costs per kilogram. A varied industry with numerous varieties could also help curb the spread of diseases and pests, ensuring long-term sustainability. These goals align with the objectives of the California Avocado Breeding Program at UC Riverside (UCR) [10].

Avocado fruit quality is strongly influenced by fungal diseases, particularly postharvest diseases that originate from infections occurring both before and during harvest. Lesions are occasionally observed in the field, but most fruits remain symptomless, with latent infections that manifest as rots during ripening [11–14]. These postharvest rots are typically classified into two categories: stem-end rot and body rot [13]. Stem-end rot initially appears as a dark brown to black lesion at the fruit’s peduncle (stem-end), often accompanied by dark streaks in the internal vascular bundles, a condition referred to as vascular streaking. As the fruit ripens, this rot progresses, resulting in decay, overall discoloration, brown flesh, and softening [13,15]. Body rots can develop at any part of the fruit, appearing as discrete, often firm, symmetrical areas of discolored flesh [16]. Both diseases can be caused by a wide range of fungi, with the most common pathogens including species in the Botryosphaeriaceae family and the genus *Colletotrichum*. *Diaporthe* species are also implicated, though they are seldom isolated from body rots [9,13,17,18]. The predominance of fungal species varies with the environmental conditions in different avocado-growing regions [13] and is further influenced by the co-occurrence of other diseases. Interestingly, fungal pathogens associated with branch canker and twig dieback can serve as sources of inoculum for fruit rot, while fungi responsible for fruit rot can also cause branch canker and twig dieback [18–21]. Understanding the identity of these pathogens and the timing of their infections in the field is crucial for determining effective management interventions.

An exceptional characteristic of avocado biology is its unique maturation process. Unlike most fruits, avocados only ripen after harvest and can remain on the tree for over 12 months after reaching physiological maturity. This flexibility in harvest timing allows horticultural maturity to be controlled, enabling marketers to coordinate harvest with periods of high demand [22]. However, maturity criteria must be followed, as they directly impact eating quality [3,22,23,24]. Consequently, dry matter percentage (DM) is used as an indicator of avocado maturity, and California minimum maturity standards are based on DM thresholds established to ensure acceptable fruit quality, such as 20.8% for ‘Hass’ [25]. These thresholds are largely based on studies showing strong correlations between DM and sensory quality attributes such as flavor and texture [26,27], although standards remain undefined for many varieties.

Fruit maturity, and consequently harvest timing, plays a critical role in determining avocado quality, and postharvest shelf life. Numerous studies, mainly focused on ‘Hass’ avocados, have investigated its direct effects on various aspects, including fruit decay [15,16,22,24]. Fruits generally become more susceptible to fungal diseases as they age on the tree and DM increases. Similarly, higher risks for disease development are expected in extended storage periods [16]. However, the inherent susceptibility of the variety plays a pivotal role in determining the final level of disease [28]. Elucidating the influence of these factors and their possible interactions is crucial to the development of management strategies, particularly for less commonly cultivated varieties.

Given this background, this study aimed to investigate the influence of avocado maturity, storage period, and variety on the occurrence of fungal diseases. The specific objectives were to: a) determine the DM range for each variety throughout the season; b) assess the severity of stem-end and body rot at different harvest times, corresponding to different maturity stages; c) identify and characterize the most common fungal species causing fruit symptoms and to assess the impact of storage period on their frequencies; and d) examine seasonal variation in the inherent susceptibility of various avocado varieties to fungal infections.

## Materials and methods

### Varieties and selections

This study included several avocado varieties and breeding selections relevant to California production systems. Commercial cultivars included ‘Hass’, the dominant global cultivar and reference standard for maturity and dry matter characterization, and ‘GEM’, a Hass-related cultivar developed through the UCR breeding program [2,29]. Two cultivars, ‘Eugenin’ and ‘Flavia’, are stable somatic mutations derived from ‘Hass’ that originated in commercial orchards in Chile [30,31]. ‘Luna UCR’ originates from the UCR avocado breeding program and derives from open-pollinated ‘Gwen’, which is a descendant of ‘Thille’, with ‘Hass’ as the great-grandparent cultivar [32]. Additional plant material consisted of advanced, unreleased breeding selections from the UCR program, including I27EG, I55B, I49LB, I54J, and I73WL. Together, these cultivars and selections encompassed substantial diversity in harvest windows, dry matter accumulation patterns, and postharvest behavior, enabling a comprehensive evaluation of maturity dynamics and disease susceptibility.

### Avocado fruit collection and dry matter determination

Avocado fruits were harvested from trees situated within the experimental area at the University of California South Coast Research and Extension Center in Irvine, CA (33° 41′ 22.79″ N, 117° 43′ 13.45″ W). At the initiation of this study, trees were 10 years old, grafted onto Dusa clonal rootstock, and grown in a Hanford sandy loam soil. Standard fertilization practices for California avocado production were followed throughout the study, and no insecticide or fungicide applications were made. Each variety was harvested multiple times over three avocado seasons in California, as detailed in Table 1. Unreleased selections, together with ‘Luna UCR’, ‘Flavia’, and ‘Eugenin’, were sampled within their expected harvest windows, with harvests scheduled based on fruit availability and to represent early-, mid-, and late-season maturity stages. ‘Hass’ and ‘GEM’, which have established minimum maturity standards, were sampled throughout their respective harvest seasons from early to late maturity. A total of 250 fruits were consistently collected from a designated set of 8–10 trees per variety during each harvest. After collection, the fruits were transported in a climate-controlled vehicle to the Kearney Agricultural Research and Extension Center (KARE) in Parlier, CA (36° 36′ 03.08″ N, 119° 30′ 34.49″ W), which is approximately a 4.5-h drive. Upon arrival, samples were stored overnight at 5 °C and 90% relative humidity. The following morning, the avocados were examined and those displaying external defects were discarded. The dry matter percentage was determined for each harvested variety using a random selection of 15 fruits. For each fruit, a single tissue plug (approximately 1 cm^3^) was extracted from both sides of the equatorial region using the Hofshi coring system, resulting in a total of two plugs per fruit. These plugs were dried using a food dehydrator (NESCO FD-75PR, Two Rivers, WI) at 65 °C for 48 h, and dry matter percentages were calculated following the procedure outlined by Arpaia et al. (2001).

**Table 1:** Harvests from October (month 10) to August (month 08) for varieties and selections across three avocado seasons (2020/2021, 2021/2022, 2022/2023).

Variety/Selection^a^	Season	Month										
		10	11	12	1	2	3	4	5	6	7	8
Hass	2020/2021					x				x		x
	2021/2022		x	x	x	x	x	x	x	x		x
	2022/2023	x	x		x		x		x	x		x
GEM	2020/2021									x		x
	2021/2022							x	x	x		
	2022/2023				x		x		x	x		x
Luna UCR	2020/2021					x				x		x
	2021/2022				x		x	x		x		x
	2022/2023				x				x	x		x
Eugenin	2020/2021											
	2021/2022		x	x	x	x	x	x	x			
	2022/2023	x	x									
Flavia	2020/2021											
	2021/2022		x	x	x	x	x	x	x			
	2022/2023	x	x									
I27EG	2020/2021											
	2021/2022		x	x								
	2022/2023	x	x		x							
I55B	2020/2021					x						
	2021/2022		x	x	x	x	x					
	2022/2023		x		x		x					
I49LB	2020/2021					x						
	2021/2022		x	x	x	x	x					
	2022/2023											
I54J	2020/2021					x				x		x
	2021/2022					x		x	x	x		x
	2022/2023						x		x	x		x
I73WL	2020/2021											
	2021/2022							x		x		x
	2022/2023								x			

^a^Entries with codes represent advanced selections from the University of California, Riverside breeding program and are not registered commercial varieties.

### Fruit storage

Avocados underwent various durations of cold storage to simulate potential market scenarios. At each harvest time, fruits from each harvested variety were randomly selected and assigned to three fiberboard cartons, each containing 25 fruits. Each carton corresponded to one cold-storage duration: 1, 3, or 6 weeks. The cartons were stored at 5 °C and 90% relative humidity, and one carton per variety was removed after each storage period.

### Disease evaluation

Following the respective cold storage period, fruits were held at 20 °C with 90% RH to allow ripening. Fruits were considered ripe when they reached optimum eating firmness (4.4–6.7 N) [23], measured using a penetrometer fitted with an 8-mm tip (Imada, Northbrook, IL, USA). After firmness measurement, all fruits were longitudinal cut and peeled to facilitate the evaluation of fungi-caused common diseases and disorders, following the guidelines outlined by White et al. (2009). The assessed diseases included stem-end rot and body rot, and the vascular streaking disorder. Disease severities were evaluated using pre-established rating scales [24], with slight modifications. For stem-end rot, disease severity was assessed on a scale from 0 to 3, interpreted as follows: 0 = no visible symptoms; 1 = 1–10%; 2 = 11–33%; and 3 = > 33% of fruit decayed. Regarding body rot and vascular streaking, a scale ranging from 0 to 5 was employed, with interpretations as follows: 0 = no visible symptoms; 1 = 1–20%; 2 = 21–40%; 3 = 41–60%; 4 = 61–80%; and 5 = > 80%.

### Fungal isolation

Fruits exhibiting symptoms of either stem-end rot (*n* = 155) or body rot (*n* = 358), or both, were collected from various varieties, storage periods, and harvest times. These symptomatic fruits were individually placed in sterile bags and used for subsequent fungal isolations. Pathogens were isolated by transferring a fragment of diseased flesh from the lesion margin onto Petri plates containing potato dextrose agar (PDA; Difco Laboratories, Detroit, MI). The PDA was supplemented with lactic acid (10 ml of 25% vol/vol per liter) to inhibit bacterial growth. The plates were then incubated at 25 °C in darkness for 5–7 days. Subsequently, mycelial plugs obtained from the actively growing margins of the colonies were transferred to fresh PDA plates for subculturing. The isolates were then examined for colony color, growth pattern, as well as the presence and morphology of reproductive structures. Based on their morphological characteristics, the isolates were grouped, allowing for preliminary identification of pathogens at the genus level.

### Molecular characterization

Following initial characterization, 23 representative isolates of Botryosphaeriaceae, 20 of *Colletotrichum* spp*.*, and 22 of *Diaporthe* spp. were selected for molecular characterization due to the predominance of their respective morphological groups and their association with stem-end rot and body rot.

### DNA extraction and amplification

Pure cultures of Botryosphaeriaceae and *Diaporthe* were obtained by transferring mycelial tips from the colony margins onto fresh PDA, while single-spore cultures of *Colletotrichum* were obtained following the procedure described by Camiletti et al. [33]. For DNA extraction, mycelium and conidia were collected from cultures grown on Potato Dextrose Broth (BD, Franklin Lakes, NJ) at 25°C in darkness for 7 days. The FastDNA kit and FastPrep Instrument (MP Biomedicals, Santa Ana, CA) were used according to the manufacturer’s instructions to extract the genomic DNA. The concentration and purity of the DNA template were evaluated using a NanoDrop 2000c spectrophotometer (Thermo Fisher Scientific, Wilmington, DE) and adjusted to a concentration of 5–10 ng/mL. Three different genomic regions from Botryosphaeriaceae and *Diaporthe* were amplified, including the internal transcribed spacer ITS1-5.8S-ITS2 (ITS), beta-tubulin (*tub2*), and translational elongation factor 1-alpha (*tef1-α*). The primer pairs used were ITS1/ITS4 [34] for ITS, Bt2a/Bt2b [35] for *tub2*, and EF1-728F/EF1-986R [36] for *tef1-α*. For *Colletotrichum*, partial sequences of the glyceraldehyde-3-phosphate dehydrogenase gene (GAPDH) and the mating type gene *MAT1–2* were amplified using the primer pairs GDF1/GDR1 [37] and CgDL_F6/CgMAT1_F2 [38], respectively. Additionally, the *tub2* region was amplified using the primer pair T1/Bt2b [34]. Amplifications were carried out using a CFX96 Touch Real-Time PCR Detection System (Bio-Rad Laboratories, Hercules, CA). Each reaction mixture comprised 12.5 µl of SYBR Green qPCR Master Mix (Bio-Rad Laboratories), 0.75 µl of each primer, 2 µl of template DNA, and 9 µl of nuclease-free water. The amplification conditions varied depending on the target region and organism. For Botryosphaeriaceae and *Diaporthe* gene regions, the reaction conditions were set as follows: an initial preheat at 95 °C for 4 minutes, followed by 40 cycles of denaturation at 95 °C for 30 seconds, annealing at 58 °C for 30 seconds, extension at 72 °C for 30 seconds, and a final elongation step at 72 °C for 5 minutes. For the amplification of the *MAT1–2* region in *Colletotrichum*, the annealing and extension times were increased to 45 s and 1 min, respectively, with a final elongation step of 10 min. The amplification conditions and mixtures for the tub2 and GAPDH regions in Colletotrichum were adjusted to match those described in Camiletti et al. (2022). The PCR products underwent purification using ExoSAP-IT (Affymatrix Inc., Santa Clara, CA) following the manufacturer’s instructions. Subsequently, the purified products were submitted to the University of California Davis sequencing facility for bidirectional sequencing.

### Multilocus phylogenetic analysis

The sequences obtained in this study were compared against the GenBank database, with a specific focus on type materials, to initially identify the closest matching species and species complexes. In particular, the *C. gloeosporioides* species complex was identified, and relevant sequences from publications available in GenBank were retrieved [38,39]. A similar approach was followed for the *Diaporthe* representative isolates, and sequences of species in the *D. oncostoma* species complex were retrieved. To compare the sequences of the Botryosphaeriaceae representative isolates selected in this study, 65 reference sequences were selected based on recent literature [40,41]. Previous studies [39,42–44] have demonstrated the compatibility of the selected genomic regions used in this study. Therefore, a combined dataset was created by aligning and manually adjusting the sequences from isolates belonging to the same genus using the MAFFT v7 online server (https://mafft.cbrc.jp/alignment/server/large.html) [45] and MEGA7 software [46] as necessary.

Phylogenetic analyses were conducted separately for the *C. gloeosporioides* species complex, the *D. oncostoma* species complex, and the *Neofusicoccum* genus. Two methods were used for *Colletotrichum* and *Diaporthe*: maximum likelihood (ML) analysis via IQ-TREE and Bayesian inference (BI) analysis using MrBayes v3.2.6 [47]. *C. xanthorrhoeae* and *Cytospora disciformis* were included as outgroups for *Colletotrichum* and *Diaporthe*, respectively. The best-fit substitution model for each species complex was determined based on the Akaike information criterion corrected using ModelFinder [48]. For the ML analysis, likelihood scores were simulated until convergence, and statistical support was estimated using an ultrafast bootstrap approach (UFBoot) with 1,000 pseudoreplicates. Zero branch length nodes collapsed. The BI analysis involved running two parallel runs with sampling every 10,000 generations, and convergence was considered achieved when split frequencies reached a value less than or equal to 0.01. In both analyses, the first 25% of the generated trees were discarded as burn-in. The 50% majority consensus trees were calculated for the ML analysis, and posterior probability values were obtained for the BI analysis. The resulting trees from the three analyses were visualized using FigTree v. 1.4.3 (http://tree.bio.ed.ac.uk/software/figtree/), and their topologies were compared. Incongruences and conflicts between clades with significant posterior probability/bootstrap support were assessed and compared with previously published phylogenies [39–41,44]. Clades were considered valid when the posterior probability values exceeded 0.9 for the BI analysis and when the bootstrap values were above 80% for the ML analysis.

Regarding the *Neofusicoccum* spp. phylogenetic analyses, three approaches were used: Maximum Parsimony (MP) analysis, ML, and BI. MP analysis was performed in PAUP v.4.0a [49] using heuristic search and tree bisection and reconstruction (TBR) as branch swapping algorithms with the branch swapping option set on ‘best trees’ only. Gaps were treated as ‘missing,’ characters were unordered and of equal weight, and Maxtrees were limited to 100. Tree scores including: Tree length (TL), Consistency Index (CI), Retention Index (RI), and Rescaled Consistency Index (RC) were calculated. The AIC was employed to identify the best-fit model of nucleotide evolution for each gene using MrModeltest v. 2.4 [50]. The ML of the combined genes was performed in GARLI v.0.951 [51]. For both analyses, clade support was assessed by 1,000 bootstrap replicates. The Bayesian analysis was conducted as described above. The *Botryosphaeria dothidea* isolates CMW8000 and CBS 110302 served as the outgroup. All sequences obtained in this study have been deposited in GenBank (Tables 2–4).

**Table 2:** Sequences of *Colletotrichum* isolates from this study used in phylogenetic analyses and deposited in GenBank.

Species	Isolate ID	GAPDH	*tub2*	*MAT1–2*
*Colletotrichum perseae*	15J01	PQ570440	PQ565786	PQ565765
	15J58	PQ570442	PQ565789	PQ565766
	15J59	PQ570445	PQ565806	PQ565767
	15J61	PQ570446	PQ565790	PQ565768
	15J63	PQ570447	PQ565787	PQ565769
	15J64	PQ570448	PQ565791	PQ565770
	15J65	PQ570449	PQ565792	PQ565771
	15J68	PQ570450	PQ565788	PQ565772
	15J82	PQ570452	PQ565793	PQ565773
	15J83	PQ570453	PQ565799	PQ565774
	15J84	PQ570454	PQ565794	PQ565775
	15J85	PQ570444	PQ565805	PQ565776
	15J86	PQ570441	–	PQ565777
	15J87	PQ570443	PQ565803	PQ565778
	15J88	–	PQ565795	PQ565779
	15J89	PQ570455	PQ565802	PQ565780
	15J90	PQ570456	PQ565800	PQ565781
	15J91	PQ570457	PQ565801	PQ565782
	15J92	PQ570458	PQ565796	PQ565783
	15J93	PQ570459	PQ565797	PQ565784
	15J94	PQ570460	PQ565798	PQ565785
	15J70	PQ570451	PQ565804	*–*

**Table 3:** Sequences of Botryosphaeriaceae isolates from this study used in phylogenetic analyses and deposited in GenBank.

Species	Isolate ID	ITS	*tef1-α*	*tub2*
*Neofusicoccum australe*	B11	PQ151756	PQ177903	PQ177924
*N. luteum*	B3	PQ151757	PQ177904	PQ177925
	B6	PQ151758	PQ177905	PQ177926
	B7	PQ151759	PQ177906	PQ177927
	B8	PQ151760	PQ177907	PQ177928
	B10	PQ151761	PQ177908	PQ177929
	B16	PQ151762	PQ177909	PQ177930
	B18	PQ151763	PQ177910	PQ177931
	B24	PQ151764	PQ177911	PQ177932
*N. nonquaesitum*	78	PQ151765	PQ177912	PQ177933
	B2	PQ151766	PQ177913	PQ177934
	B5	PQ151767	PQ177914	PQ177935
	B12	PQ151768	PQ177915	PQ177936
	B13	PQ151769	PQ177916	PQ177937
	B14	PQ151770	PQ177917	PQ177938
	B15	PQ151771	PQ177918	PQ177939
	B17	PQ151772	PQ177919	PQ177940
	B21	PQ151773	PQ177920	PQ177941
	B22	PQ151774	PQ177921	PQ177942
*N. parvum*	B9	PQ151775	PQ177922	PQ177943
	B19	PQ151776	PQ177923	PQ177944

**Table 4:** Sequences of *Diaporthe* isolates from this study used in phylogenetic analyses and deposited in GenBank.

Species	Isolate ID	ITS	*tef1-α*	*tub2*
*Diaporthe baccae*	P13	PQ357364	PQ570461	PQ570462
*D. foeniculina*	P1	PQ357351	PQ565750	PQ565763
	P2	PQ357354	PQ565739	PQ565762
	P3	PQ357355	PQ565746	PQ565764
	P6	PQ357350	PQ565744	PQ565752
	P7	PQ357357	PQ565749	PQ565759
	P8	PQ357359	PQ565748	PQ565761
	P9	PQ357352	PQ565747	PQ565753
	P15	PQ357353	PQ565737	PQ565754
	P16	PQ357363	PQ565738	PQ565755
	P18	PQ357356	PQ565740	PQ565751
	P19	PQ357360	PQ565745	PQ565757
	P20	PQ357358	PQ565741	PQ565756
	P21	PQ357362	PQ565743	PQ565758
	P22	PQ357361	PQ565742	PQ565760

### Susceptibility tests

Experiments were conducted to investigate seasonal variations in inherent susceptibility to *Neofusicoccum*, *Diaporthe*, and *Colletotrichum* in each harvested variety. At each harvest time, 36 fruits were selected and stored in a cold room at 5 °C and 90% RH until further use but not exceeding three days. The unripe fruits were surface disinfected prior to the experiments by immersing them in a 0.6% NaOCl solution (10% Clorox bleach; The Clorox Company, Oakland, CA) for 4 minutes. Subsequently, the fruits were air-dried on a sterile surface within a laminar flow hood for 30 minutes. The disinfected fruits were placed on raised plastic mesh inside humid plastic chambers (30 cm × 21 cm × 10 cm), which had been previously sterilized using a 0.6% NaOCl solution. Each chamber accommodated six fruits. Inoculations were conducted utilizing three pathogens: *N. nonquaesitum* (isolate B78), *D. foeniculina* (isolate P15), and *C. perseae* (isolate 15J01). The selected pathogens corresponded to the most prevalent species identified during the surveys conducted in this study. Using a 6-mm-diameter cork borer, a hole approximately 2–3 mm deep was made on the equatorial region of each fruit. Subsequently, mycelial agar discs, measuring 6 mm in diameter, were obtained from actively growing cultures and placed upside down into the holes. To prevent contamination and dehydration, the discs were covered with petroleum jelly. Additionally, an extra humid chamber containing fruits inoculated with agar plugs (without the pathogen) was prepared as control. Approximately 200 ml of tap water was added underneath the mesh in each chamber to maintain high humidity. All treatments were incubated at an approximate temperature of 25°C. The experiment was conducted in a completely randomized design, with six replications (six fruits per humid chamber) for each combination of pathogen and variety. The experiment was conducted twice for each variety at each harvest time. Fruits inoculated with *N. nonquaesitum* were evaluated after 7 days, while the remaining treatments were assessed after 10 days of incubation. At the time of evaluation, all fruits had reached full ripeness as determined by visual assessment. Fruits were then peeled, and the length of the internal lesion was measured twice in perpendicular directions using a digital caliper.

### Statistical analysis

An initial chi-squared test of independence (α = 0.05) was conducted to investigate the association between symptom types and pathogen frequencies. Subsequently, paired chi-squared tests (α = 0.05) were separately performed for each symptom type to compare the frequencies of individual pathogens. Chi-square tests were also conducted to explore the effect of the storage period on pathogen frequencies.

Ordinal data from severity evaluations were analyzed using cumulative link models with a logit link, also known as proportional odds models [52]. These models have shown superiority to the midpoint conversion of the interval method, especially at low disease severity [53]. Models were fitted separately for each avocado variety, including the variable month as a fixed effect, and using the ‘clm’ function of the ‘ordinal’ R package [52]. The significance of the variable was assessed by performing a likelihood ratio test using the ‘lrtest’ function from the ‘lmtest’ R package. Means were estimated using the ‘emmeans’ function from the ‘emmeans’ R package [54], while all pairwise comparisons were conducted with the ‘cld’ function of the ‘multcomp’ R package (α = 0.05).

Data from inoculation experiments were subjected to analysis using linear models with harvest month and variety as fixed effects. This analysis was performed using the ‘lm’ function from the ‘stats’ R package. Subsequently, the significance of variables and interactions was further assessed utilizing the ‘anova’ function within the same package. Means were then estimated and compared as previously described.

## Results

### Seasonal variation of dry matter percentages

**Fig 1:**
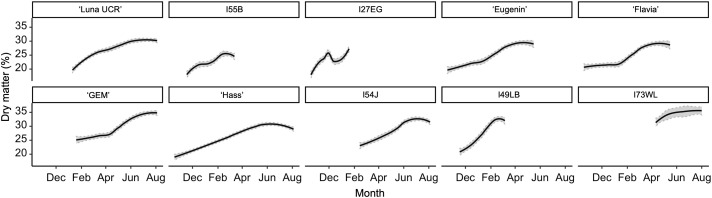
Seasonal variation of dry matter percentages for each variety using data collected during three consecutive seasons.

The seasonal variation in the percentage of dry matter for each variety is displayed in Fig 1. In the case of ‘Hass’, the percentage of dry matter steadily increased from the first harvest, quickly reaching its minimum maturity standard (20.8%) by December. The peak percentages were observed in June, with a slight decline noted in August. Similarly, the varieties ‘GEM’, I54J, and ‘Luna UCR’ exhibited comparable seasonal trend, with maximum DM recorded in June. The varieties ‘Eugenin’ and ‘Flavia’ followed the same pattern, though their peak percentages were recorded earlier, specifically in April. For I27EG and I55B, the maximum percentages occurred in January and February, respectively, with mean values relatively lower than the other varieties. The variety I49LB also showed peak percentages in February. In contrast, the variety I73WL maintained relatively high percentages throughout the three harvests, with mean values exceeding 30%.

### Seasonal variations in severity scores for body rot, stem end rot, and vascular streaking

**Fig 2:**
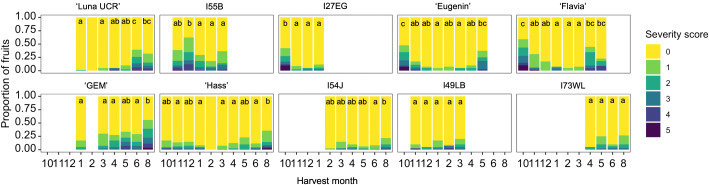
Distribution of body rot severity scores for avocado varieties harvested from October to August. Different letters indicate significant differences among harvest months within each variety based on Tukey-adjusted pairwise comparisons (*P* < 0.05).

The distribution of severity scores for body rot throughout the year in each avocado variety is shown in Fig 2. Distinct seasonal patterns were observed among the genotypes. Varieties ‘Eugenin’ and ‘Flavia’ exhibited a similar trend, with the highest severity scores observed at the beginning and end of the harvesting season. ‘Luna UCR’, ‘GEM’, and I54J also shared a similar pattern, where severity remained low during the first harvest but increased towards the end of the season. Conversely, varieties I27EG and I55B displayed the opposite trend, with the highest severity during the first harvests. It was difficult to describe a specific pattern in the cultivar ‘Hass’, although the highest severity was observed in the August harvest. Finally, I73WL and I49LB exhibited relatively low severity of body rot, unaffected statistically by harvest time.

**Fig 3:**
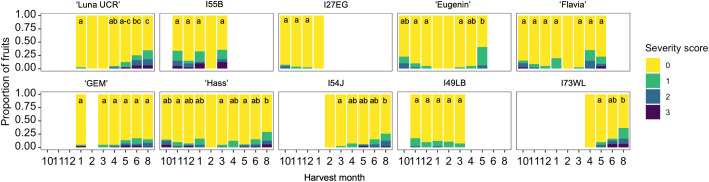
Distribution of stem-end rot severity scores for avocado varieties harvested from October to August. Different letters indicate significant differences among harvest months within each variety based on Tukey-adjusted pairwise comparisons (*P* < 0.05).

The severity of stem end rot generally exhibited similar trends to body rot (Fig 3), although the differences were less pronounced. In fact, in ‘Flavia’, I27EG, I55B, ‘GEM’, and I49LB, the severity of stem end rot symptoms did not vary statistically due to harvest time. ‘Eugenin’ displayed the highest severity scores at the beginning and end of the season. ‘Luna UCR’, I73WL, and I54J exhibited the highest severity scores towards the end of the season. Determining a pattern in ‘Hass’ remained challenging, although again, the highest severity was observed in August, at the end of its season.

**Fig 4:**
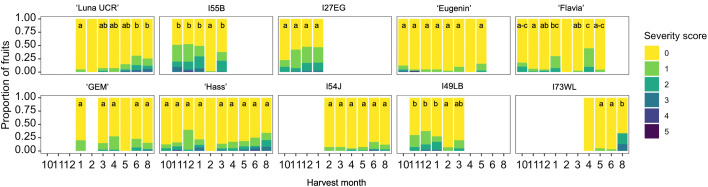
Distribution of vascular streaking severity scores for avocado varieties harvest from October to August. Different letters indicate significant differences among harvest months within each variety based on Tukey-adjusted pairwise comparisons (*P* < 0.05).

The seasonal patterns of vascular streaking severity for each avocado variety are presented in Fig. 4. Statistical analysis revealed no significant variations among harvest months for the varieties ‘Eugenin’, ‘Hass’, I27EG, ‘GEM’, and I54J. In contrast, ‘Luna UCR’ and I73WL exhibited the highest severity values in the latest harvest, while I49LB showed the lowest scores during this period. I55B recorded the lowest severity scores during the second harvest. Although ‘Flavia’ exhibited variations, a clear pattern was challenging to elucidate.

### Pathogen frequency

**Fig 5:**
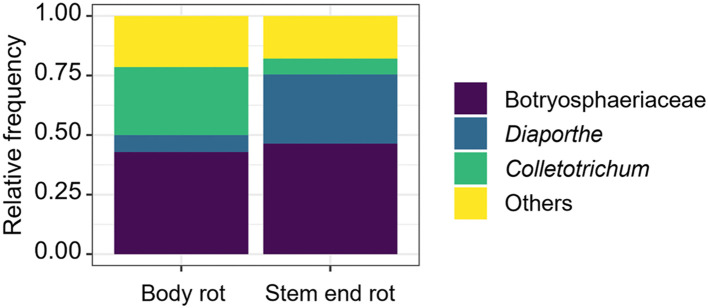
Distribution of fungal groups isolated from avocado fruits with symptoms of stem end rot and body rot.

The analyzed fruit samples comprised the avocado varieties I55B (*n* = 23), ‘GEM’ (*n* = 125), ‘Hass’ (*n* = 109), I54J (*n* = 76), ‘Luna UCR’ (*n* = 137), and I73WL (*n* = 43), as well as samples after 1 week (*n* = 62), 3 weeks (*n* = 154), and 6 weeks (*n* = 297) of cold storage. Most of the isolates obtained from the symptomatic flesh exhibited morphological characteristics consistent with the descriptions provided by Twizeyimana et al. (2013) [18] for Botryosphaeriaceae, *Colletotrichum* spp., and *Diaporthe* spp., which affect avocado fruits in California. The relative frequency of these three fungal groups was influenced by the disease (Chi-square = 57, df = 2, *P* < 0.001), as observed in Fig 5. Among the 358 fruits displaying body-rot symptoms across all varieties, Botryosphaeriaceae had higher frequencies than *Colletotrichum* spp. (Chi-square = 9.2, df = 1, *P* = 0.002), while *Diaporthe* spp. occurred less frequently (Chi-square = 41, df = 1, *P* < 0.001). Similarly, among the 155 fruit with stem-end rot symptoms, Botryosphaeriaceae were also the most predominant group and had frequencies higher than *Diaporthe* spp. (Chi-square = 5.9, df = 1, *P* = 0.01). *Colletotrichum* spp. were recovered at a very low frequency and were primarily associated with fruit that also exhibited body rot symptoms. Additional identified fungi included species of *Alternaria*, *Cladosporium*, *Penicillium*, as well as other unidentified morphotypes.

### Multilocus phylogenetic analyses

**Fig 6:**
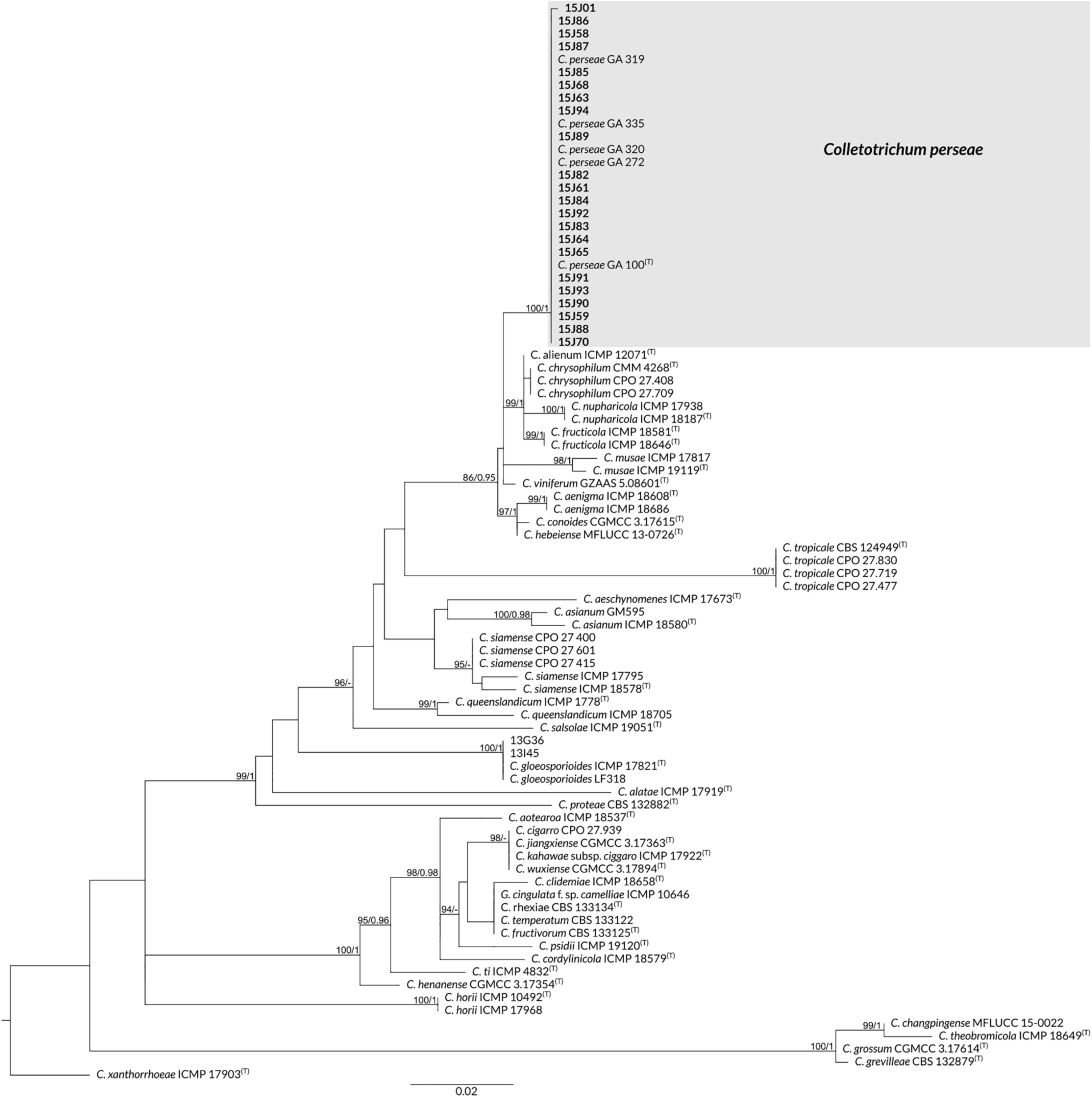
A phylogenetic tree based on Bayesian inference for the *Colletotrichum gloeosporioides* species complex was created. This tree was constructed utilizing concatenated sequences from the GAPDH, *tub2*, and *MAT1-2* regions. At each node, bootstrap support values exceeding 80% from maximum likelihood (ML) and posterior values greater than 0.9 from Bayesian inference (BI) are indicated (ML/BI). *C. xanthorrhoeae* served as the outgroup. Isolates from this study are indicated in bold.

The representative isolates of *Colletotrichum* belonged to the *C. gloeosporioides* species complex. The final data matrix consisted of 796 characters with gaps (GAPDH: 1–190, *tub2*: 191–475, and *MAT1–2*: 476–796). For BI and ML analyses, the selected model was GTR + F + I for the three genes. The consensus tree obtained from ML analysis conﬁrmed the tree topology obtained with BI. Ultrafast bootstrap support values agreed with Bayesian posterior probability. In the multilocus phylogeny, the 22 representative isolates clustered with reference strains of *Colletotrichum perseae* previously isolated from avocados in Israel, forming a well-supported and distinct clade (BI/ML = 1/100), as shown in Fig 6.

**Fig 7:**
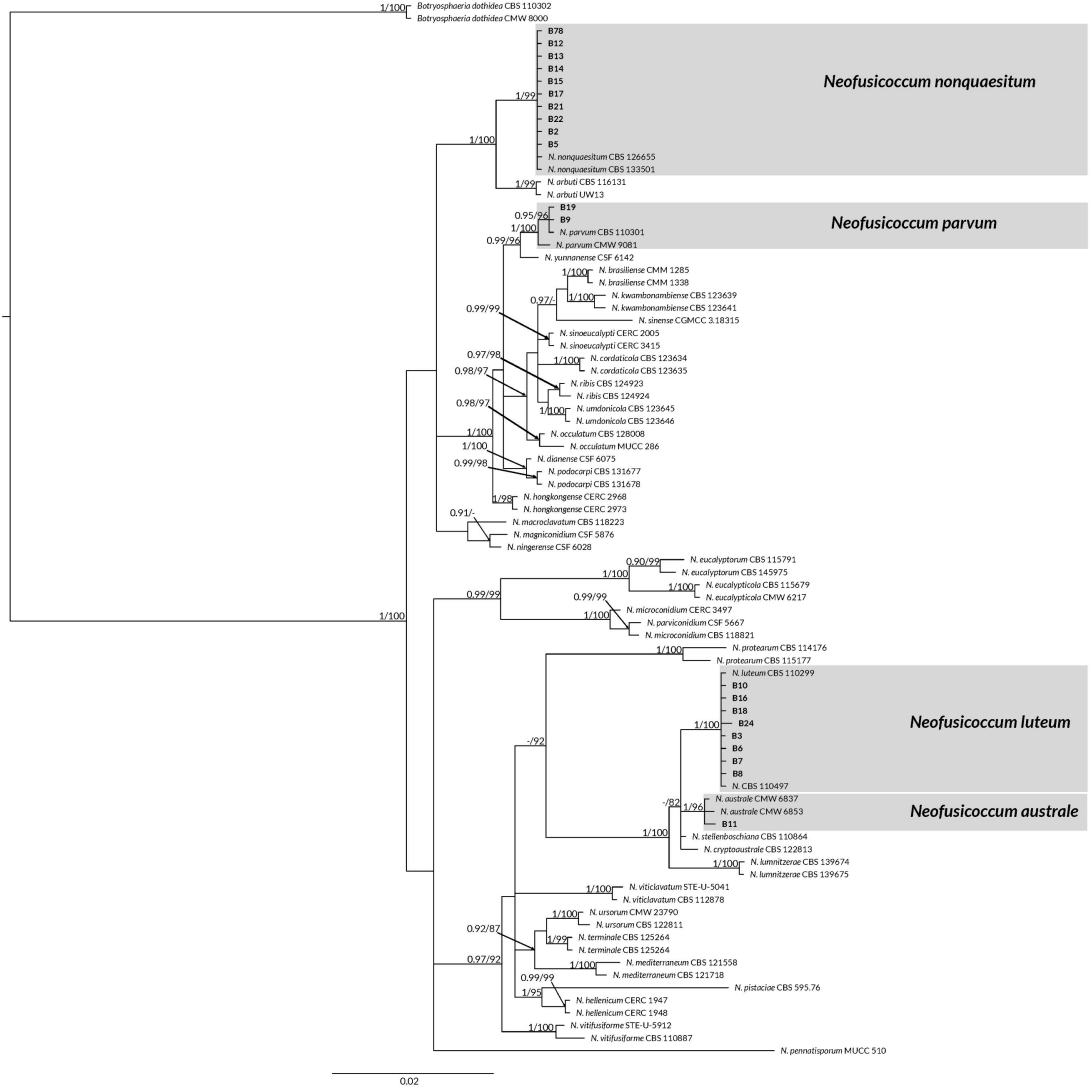
A Bayesian inference phylogenetic tree of Botryiosphaeria species. The phylogenetic tree was built using concatenated sequences of the ITS, *tub2*, and *tef1-α* regions. Bayesian inference (BI) posterior values more than 0.9 and bootstrap support values from maximum likelihood (ML) more than 80% are shown at each node (BI/ML). *Botryosphaeria dothidea* isolates CMW8000 and CBS 110302 were used as the outgroup. Isolates from this study are shown in bold.

Regarding the Botryosphaeriaceae analyses, the MP tree, ML tree, and Bayesian tree showed consistent topologies. In the MP analysis, the combined dataset revealed that out of 1,876 total characters, 250 were parsimony-informative, 88 were parsimony-uninformative, and 1,538 were constant. A total of 100 trees were retained. Tree length was equal to 584, CI = 0.676, RI = 0.908, RC = 0.614. The best-fit model of nucleotide evolution based on the AIC was SYM + I + G for ITS, and HKY + G for *tef1*-α and *tub2*. The ML analysis revealed that out of 1,876 total characters, 1,538 were constant, 287 were parsimony-informative, and 51 were autapomorphic. Isolates B9 and B19 strongly clustered within the clade of *N. parvum* (97/92/1 for MP, ML bootstrap support %, and Bayesian posterior probability, respectively). Additionally, isolates B78, B2, B5, B12, B13, B14, B15, B17, B21, and B22 strongly clustered within the clade of *N. nonquaesitum* (99/90/1), while isolates B3, B6, B7, B8, B10, B16, B18, and B24 clustered within the *N. luteum* clade (99/99/1). Finally, isolate B11 grouped within *N. australe* clade (84/78/1). Based on our analyses, the Botryosphaeriaceae isolates were identified as *N. australe*, *N. luteum*, *N. nonquaesitum* and *N. parvum* (Fig 7).

**Fig 8:**
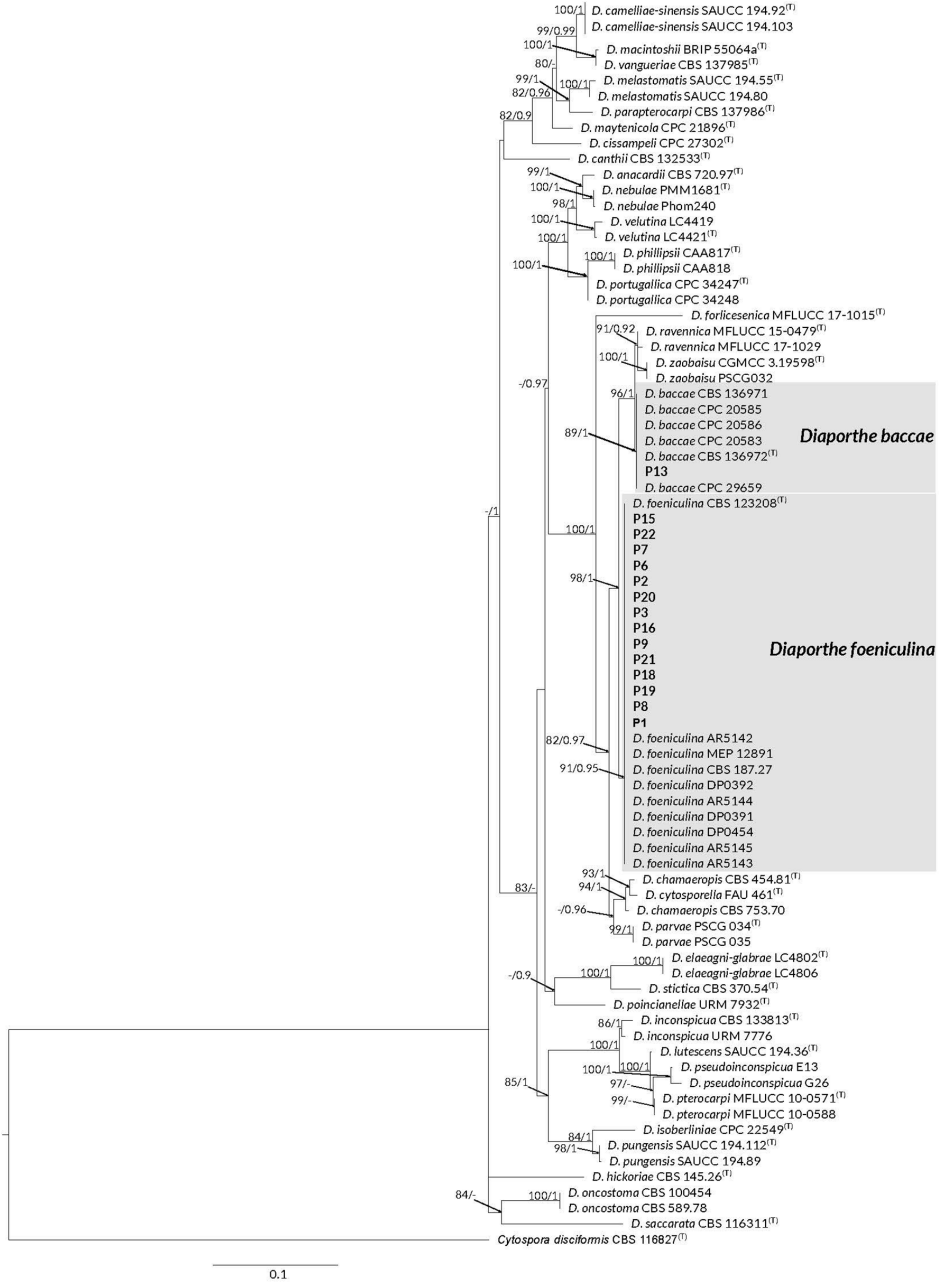
A phylogenetic tree of the *D. oncostoma* species complex using Bayesian inference. The ITS, *tub2*, and *tef1-α* regions’ concatenated sequences were used to construct the phylogenetic tree. Each node (ML/BI) displays bootstrap support values from maximum likelihood (ML) greater than 80% and Bayesian inference (BI) posterior values greater than 0.9. The outgroup was *Cytospora disciformis*. Isolates from this study are shown in bold.

The representative isolates of *Diaporthe* belonged to the *D. oncostoma* species complex. The final data matrix consisted of 1077 characters with gaps (ITS: 1–422, *tef1*-α: 423–718, and *tub2*: 719–1077). For BI and ML analyses, the selected model was GTR + F + I for the three genes. The consensus tree obtained from ML analysis conﬁrmed the tree topology obtained with BI. Ultrafast bootstrap values agreed with Bayesian posterior probability. One of the isolates grouped with type material of *D. baccae* in a distinct clade with significant statistical support (1/89 BI/ML). The rest of the representative isolates grouped with strains of *D. foeniculina* in a distinct clade with significant statistical support in the multilocus analysis (0.95/91, BI/ML), as shown in Fig 8.

### Interactions between variety and harvest timing in avocado inherent susceptibility

After the incubation period, inoculated fruits developed lesions with well-defined margins, ranging from discolored areas to dark-brown or black, typically accompanied by flesh softer than the surrounding areas (S1–S3 Fig). The intensity of color and the softness of the flesh varied, depending on the pathogens and varieties, making distinctive features difficult to determine. However, lesions caused by *C. perseae* tended to be a darker brown than lesions of other species (S2 Fig). In general, the lesion size was influenced by the pathogen (*P* < 0.001). *N. nonquaesitum* generated the largest lesions (57.2 ± 0.07 mm), which was also evidenced by the shortest incubation periods used during the experiments. *C. perseae* produced intermediate lesions (45.9 ± 0.07 mm), while *D. foeniculina* resulted in smaller lesions (36.8 ± 0.09 mm).

**Fig 9:**
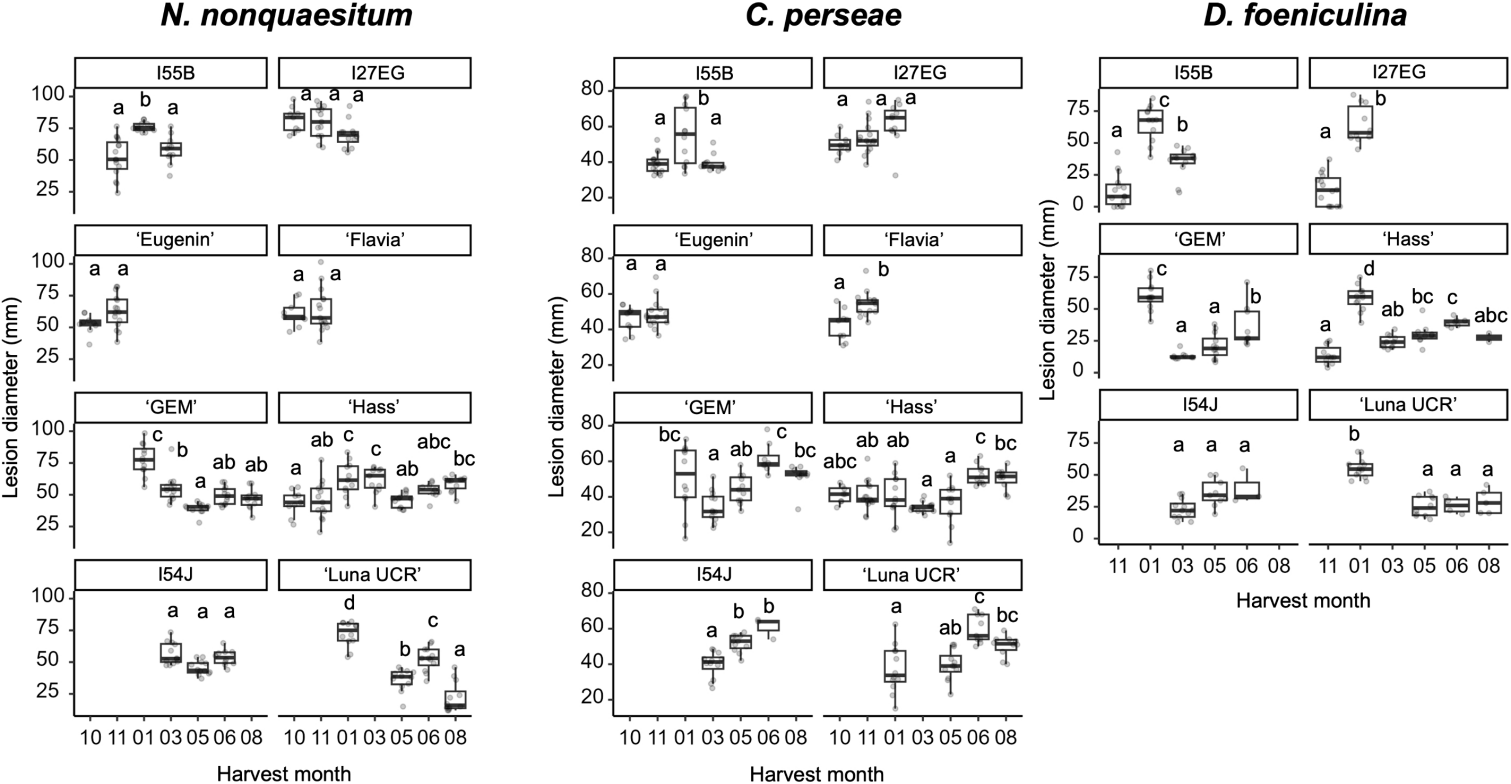
Diameter of lesions caused by stem-end rot and body rot pathogens on fruits of avocado varieties harvested from October 2022 to August 2023.

Avocado fruit susceptibility to *N. nonquaesitum* was influenced by both the specific variety and the timing of harvest, revealing a significant interaction between these variables (*P* < 0.001). Fig 9 illustrates the lesion size distributions across different harvests for each variety. Statistical analysis indicated that fruits from varieties I27EG, ‘Eugenin’, ‘Flavia’, and I54J did not exhibit significant differences in susceptibility across harvest times. However, I55B fruits displayed increased lesion sizes during January, compared to November and March. The varieties ‘GEM’, ‘Hass’, and ‘Luna UCR’ exhibited varying susceptibility levels across harvests, with notably higher lesions observed between January and March.

The response of avocado varieties to the pathogen *C. perseae* was influenced by both the specific variety and the timing of harvest, indicating a significant interaction between these variables (*P* < 0.001). The susceptibility patterns of avocado varieties over several harvests showed different trends (Fig 9). The I27EG and ‘Eugenin’ varieties displayed consistent susceptibility across harvests, with no significant differences in lesion sizes observed. I55B exhibited a significant increase in susceptibility during January. I54J and ‘Flavia’ exhibited a similar trend, with a significant increase in susceptibility during the later harvests. ‘GEM’, ‘Luna UCR’, and ‘Hass’ exhibited a similar and dynamic susceptibility pattern, with the highest lesion size observed during June.

Both harvest time and variety had a significant effect on the affected lesion size caused by *D. foeniculina*, although their interaction was not significant (*P* = 0.09). The results revealed distinct patterns of susceptibility among different avocado varieties across various harvests (Fig 9). For instance, the variety I55B exhibited a significant increase in lesion size from November to January, followed by a decline in the last harvest. Similarly, I27EG showed an increase in lesion size from November to January. ‘GEM’ displayed a notable decrease in susceptibility in March, followed by a rise in later harvests. The cultivar ‘Hass’ demonstrated varying susceptibility levels across different harvests, with the most significant increase observed in January. I54J exhibited relatively consistent susceptibility throughout the harvests. In contrast, ‘Luna UCR’ displayed a decrease in lesion size after January, remaining stable in subsequent harvests.

## Discussion

Because avocados are capable of ripening after harvest, adjusting harvest timing to capitalize on higher prices is a common practice. However, adherence to a minimum maturity standard throughout the avocado supply chain is essential to ensure a satisfactory eating fruit quality and minimize fruit disorders [16]. The use of DM as a maturity indicator is widely accepted [16,27,55]. In California, the minimum DM content ranges from 17.7% to 24.2%, depending on the variety. Among the varieties and selections studied here, ‘Hass’ and ‘GEM’ are the only ones that has been previously characterized. The minimum DM at harvest for these varieties are 20.8 and 22.8%, respectively [25]. In our study, ‘Hass’ avocados rapidly reached this standard by December, highlighting the extended harvesting season of this variety. In contrast, ‘GEM’ consistently exhibited DM values above its minimum threshold throughout the sampling period. The DM also showed a decrease towards the end of the season. While most studies on Hass have reported just a linear increase in DM throughout the season [56–58], Osuna-García et al. [59] also observed DM stabilization towards the end, with potential decreases during the final harvest, which is consistent with the DM pattern observed in our study and Camiletti et al. [Unpublished].

Seasonal DM dynamics should then be studied for new varieties or upcoming releases in California to fill gaps caused by the dominance of ‘Hass’ production. Our study is the first reporting DM patterns for eight avocado varieties and selections. Some genotypes showed similar patterns as ‘Hass’ while others differed greatly. For instance, ‘Flavia’ and ‘Eugenin’ are believed to be somatic mutations of ‘Hass’. Both displayed similar seasonal trajectories to each other, indicating shared developmental behavior; however, both diverged from ‘Hass’ in the timing and magnitude of dry matter accumulation. Such divergence is consistent with somatic variation, which can alter fruit maturation dynamics despite a common genetic background [60]. Hass-related breeding selections, including ‘GEM’ and ‘Luna UCR’, also reflected partial similarity to ‘Hass’. In contrast, cultivars with non-Hass genetic backgrounds, such as I27EG, I55B, I49LB, I54J, and I73WL, demonstrated greater variability in dry matter trajectories, including rapid early accumulation or shortened seasonal windows. These findings emphasize the importance of cultivar-specific dry matter benchmarks for optimizing harvest timing and postharvest quality.

Harvesting fruits too early in maturity can increase the occurrence of rot diseases during postharvest. Specifically, fruits are more susceptible to stem-end rot caused by fungi of the Botryosphaeriaceae family when harvested before reaching acceptable commercial maturity levels [9,26,61]. A similar pattern was observed for body rot in ‘Hass’ avocados. In response to high disease incidence, the New Zealand avocado industry adopted an average 24% DM as the minimum maturity standard for ‘Hass’ [57]. In our study, ‘Hass’ did not exhibit higher severity of body rot, stem-end rot, or vascular streaking during the first harvests. However, increased disease severities were observed in the earliest harvests for some varieties. For instance, I27EG, I55B, ‘Eugenin’, and ‘Flavia’ exhibited higher body rot severity, though these varieties did not show such marked differences for stem-end rot and vascular streaking.

There are indications that susceptibility to diseases can increase also with greater maturity [16]. Avocado anthracnose, a disease caused by species of *Colletotrichum* and strongly correlated with body rot, can be more severe in fruits harvested late in the season when the DM is higher than 29% [9,62]. Likewise, it is recommended that avocados be harvested at optimal maturity levels, as overmature fruits are more susceptible to stem-end rot during postharvest [9]. Our results showed that body rot severity increased toward the end of the season in several varieties, including ‘Hass’. This trend was also observed for stem-end rot and vascular streaking in some varieties. For instance, ‘Eugenin’, ‘Hass’, ‘Luna UCR’, and I54J exhibited higher stem-end rot severity later in the season. This could be associated with a potential reduction in the concentration of persin, an antifungal compound in the fruit skin that mediates the latency of fungal latent infections, as the fruit matures [63]. Further research is necessary to investigate the seasonal variation in the concentration of this compound in California varieties, as it could provide insights into the relative susceptibility of new varieties.

Spore trap studies conducted in California reported that spores from Botryosphaeriaceae and Diaporthaceae are released during rain events in the fall and winter, while they are present in low frequencies during the summer [64]. In New Zealand, it was found that a high proportion of stem-end infections occur during harvest, with stems infected through wounds [21]. Therefore, we expected to observe an increase in stem-end rot disease in fruits harvested during the months typically associated with heavier rains in California (December to March, Table S4 in S1 File). However, based on our results, this did not occur, or at least the severities were not higher than in fruits harvested in other months, suggesting that it might be related to a lower susceptibility of the fruits. Surprisingly, susceptibility tests with *N. nonquaesitum* and *D. foeniculina* demonstrated that fruits were, in fact, more susceptible during the rainy months, based on results from varieties such as ‘Luna UCR’, ‘GEM’, and ‘Hass’. Therefore, stem-end rot occurring postharvest in California seems to be more associated with asymptomatic endophytes in the stem tissue, which can infect stem-end tissue at a later stage, as initially suggested also by Johnson and Kotze (1994) [65]. Some additional fungal genera recovered at low frequencies, including *Alternaria*, *Cladosporium*, and *Penicillium*, may also occur as opportunistic endophytes or secondary colonizers in avocado fruit tissues, although their specific role in postharvest disease development was not investigated in this study. Species of Botryosphaeriaceae have also been identified as causing body rot, particularly in California [66]. A recent study [67] indicated that this pathogen can infect avocado fruit through lenticels under controlled conditions, yet the complete disease cycle remains unclear. In contrast, the disease cycle for *Colletotrichum*, another pathogen responsible for body rot, is well understood. In avocados, the pathogen develops an appressorium that penetrates the outer layer of the fruit skin and remains dormant due to antifungal compounds in unripe fruit. It resumes growth and causes symptoms as the fruit ripens [9,68]. *Colletotrichum* spores require extended periods of wetness to germinate [69]. Consequently, higher infection rates could occur during the winter months in California. However, given the ability of the pathogen to remain latent, this does not necessarily translate into higher body rot severities in fruits harvested in winter, as observed in this study.

As explained above, stem-end and body rots in the California environment appear to be more associated with latent infections that develop postharvest as the fruit ripens. This is also supported by the fact that symptomatic fruits were not observed throughout the surveys conducted for this study. Therefore, seasonal changes in final disease intensity could be more closely associated with variations in the inherent susceptibility of the fruits at the time of harvest. One factor could be the concentration of persin, which decreases during storage and ripening, although the degree of change depends on the concentration present at harvest [63]. The susceptibility tests in this study were conducted to confirm the relevance of the inherent susceptibility of the fruits in disease development for stem-end rots (*N. nonquaesitum* and *D. foeniculina* experiments) and body rots (*C. perseae* and *N. nonquaesitum* experiments). Results confirmed seasonal variability, although the differences among harvest months observed under controlled conditions did not always correlate with the results of fruits from the fields naturally infected over several seasons. For instance, controlled inoculation assays indicated greater susceptibility of ‘GEM’ fruit during the earliest harvest; however, this increased inherent susceptibility did not correspond to higher rot severity under natural infection conditions. Conversely, disease severity distributions in ‘Hass’ fruits showed a pattern similar to their inherent susceptibility. It should be noted that susceptibility tests were performed over one season (2022/2023), while the rot severities were collected over three seasons. It is known that the season can influence certain fruit parameters, such as DM, which will ultimately determine postharvest performance and may be responsible for such discrepancies [70,71]. In this study, all cultivars were maintained under the same standard minimum fertilization program for California avocado production, and no insecticide or fungicide applications were made during the study period; therefore, differences in susceptibility among cultivars are unlikely to be explained by cultivar-specific fertility or plant protection regimes. Additionally, *N. nonquaesitum* was more aggressive than *D. foeniculina* and *C. perseae*, as evidenced by the shorter incubation period in the experiments, being in agreement with previous studies on ‘Hass’ avocados [18]. Because only one representative isolate per species was used in the susceptibility assays, additional studies including multiple isolates per species would be valuable to determine the extent of intraspecific variability in aggressiveness and its potential influence on cultivar responses.

Understanding the primary pathogens responsible for avocado rots is crucial for effective management. Traditionally, *Colletotrichum* has been associated with body rot and considered the most economically important disease in avocado-growing regions, while Botryosphaeriaceae are known as the most common pathogens responsible for stem-end rot disease [9,17,18,72,73]. An extensive survey in California of decayed avocados over three seasons reported *Colletotrichum* and Botryosphaeriaceae as the most frequent pathogens causing stem-end rot and body rot, with their predominance influenced by season and harvest time [66]. These authors also noted that the frequency of *Colletotrichum* spp. can increase in seasons with high precipitation. Moreover, they found *Diaporthe* species in relatively low frequencies and only in late harvests [66]. More recent studies identified the isolates causing stem-end rot as Botryosphaeriaceae (65%), *Colletotrichum* spp. (33%), and *Diaporthe* spp. (2%) [18]. In our study, Botryosphaeriaceae were found to be the predominant pathogens causing both diseases. *Colletotrichum* was frequent in body rot lesions but less common in stem-end lesions. Conversely, *Diaporthe* showed the opposite trend, with high frequencies in stem-end rot but low in body rot. Besides the contrasts with Twizeyimana et al. (2013) [18], it should be noted that our survey included several varieties, as in Smilanick et al. (2002) [66], and not only Hass. Furthermore, *Diaporthe* (*Phomopsis*) is considered common in stem-end rot but rare in body rot [13,21,28,74], which agrees with our results.

In this study, four Botryosphaeriaceae species were identified from decayed avocado fruits: *N. australe*, *N. luteum*, *N. nonquaesitum*, and *N. parvum*. Studies conducted in California have revealed the diversity of Botryosphaeriaceae species causing branch canker and dieback on avocado [64,75,76]. These previous works include all the species identified in this study, and it is not surprising that this group of fungi is involved in different symptoms on the same host, ranging from fruit rot to branch canker.

Several species of *Colletotrichum* have been associated with avocado anthracnose and body rot [17,77–79]. In 2017, *C. perseae* (teleomorph of *C. aenigma*) was identified as a new species associated with avocado anthracnose in Israel [42]. Since then, this species has been reported in other avocado regions [78,80]. All the isolates collected in our study were identified as *C. perseae*.

Additionally, species belonging to Diaporthaceae, *D. baccae* and *D. foeniculina*, were identified and associated with fruit rot symptoms. California ‘Hass’ avocados have been documented to suffer from stem-end rot attributed to *D. rudis* [81], but this pathogen was not identified in our study. Several *Diaporthe* species have been implicated in stem canker in California [75], while *D. foeniculina* has been known to cause avocado canker in Greece and Italy [17,82], but has not been previously reported in fruits. To our knowledge, this study is the first to report *D. baccae* and *D. foeniculina* infecting California avocado fruits. Previously, these species have been documented to cause fruit diseases in Europe [83–85]. The inoculation experiments revealed that *N. nonquaesitum* was a more aggressive pathogen compared to *D. foeniculina*, while *C. perseae* exhibited intermediate aggressiveness. These findings are consistent with studies on avocados conducted in Italy [17].

In conclusion, this study highlights the complex interactions among fruit maturity, disease susceptibility, and postharvest performance in avocado. Our findings demonstrate the importance of adhering to both minimum and potentially maximum maturity standards to minimize fruit diseases and possibly physiological disorders. Seasonal patterns of dry matter accumulation were identified for several selections, which can aid in establishing these standards. Fruit susceptibility to stem-end rot and body rot is influenced by both DM-based maturity and harvest season. The diversity of pathogens causing postharvest rot highlights the complexity involved in managing this disease. Overall, this research significantly enhances our understanding of the factors influencing the post-harvest performance of avocados and offers valuable insights for the California avocado industry. The summary framework provided in Table 5 integrates these findings by highlighting cultivar-specific dry matter patterns, observed disease trends, and preliminary harvest considerations. These findings are instrumental in establishing optimal harvest windows to enhance fruit rot management for each variety.

**Table 5:** Summary of seasonal dry matter (DM) accumulation patterns, observed postharvest disease trends, and harvest considerations for avocado cultivars and selections evaluated across the three California avocado seasons.

Variety / Selection	General DM Pattern	Early-Season Disease Risk	Late-Season Disease Risk	Main Disease Trend Observed	Suggested Harvest Consideration^a^
Hass	Gradual DM increase, peak in June	Low to moderate	Increased	Higher body rot and stem-end rot toward late harvests	Avoid very late harvests with elevated disease severity
GEM	DM consistently above maturity threshold	Low	Moderate	Higher body rot toward late harvests	Avoid very late harvests with elevated body rot severity
Luna UCR	Similar to Hass, peak in June	Low	Increased	Body rot, stem-end rot and vascular streaking increased later in season	Early and mid-season harvests may reduce disease risk
Eugenin	Earlier DM peak (April)	Increased	Increased	Higher body rot and stem-end rot at beginning and end of season	Intermediate harvest window may minimize disease severity
Flavia	Earlier DM peak (April)	Increased	Increased	Higher body rot severity early and late season	Intermediate harvest window may minimize disease severity
I27EG	Early DM peak	Increased	Low to moderate	Highest body rot severity during earliest harvests	Delay harvest beyond earliest maturity stages
I55B	Peak DM in February	Increased	Lower	Higher body rot severity during earliest harvests	Limited effect of harvest timing, although late harvest may reduce body rot.
I49LB	Peak DM in February	Low	Low	Slightly higher vascular streaking with early harvest	Broad harvest flexibility
I54J	Similar to Hass/GEM	Low	Increased	Stem-end rot increased later in season	Avoid very late harvests with elevated disease severity
I73WL	High DM throughout season	Low	Increased	Stem-end rot and vascular streaking increased later in season	Early and mid-season harvests may reduce disease risk

^a^Except for ‘Hass’ and ‘GEM’, minimum dry matter maturity thresholds have not been formally established for the evaluated cultivars and selections; therefore, harvest considerations should be interpreted as preliminary observations based on seasonal dry matter accumulation patterns and disease severity trends observed in this study.

## Supporting information

S1 Fig Lesions caused by *N. nonquaesitum* on ‘Hass’ (A), I49LB (B), and I55B (C) fruits. Images generated and captured by the authors.
(TIF)

S2 Fig Lesions caused by *C. perseae* on ‘Hass’ (A), I49LB (B), and ‘Luna UCR’ (C) fruits. Images generated and captured by the authors.
(TIF)

S3 Fig Lesions caused by *D. foeniculina* on ‘Hass’ (A), I49LB (B), and I55B (C) fruits. Images generated and captured by the authors.
(TIF)

S1 File Supplementary Tables. (DOCX)
