# Supplementary material for: Influence of fruit maturity and variety on seasonal postharvest fungal diseases and vascular streaking in California Avocados
Source: PLoS One. 2026 Jun 25;21(6):e0351752. doi: 10.1371/journal.pone.0351752 (PMC13298749; doi:10.1371/journal.pone.0351752)
Supplement: S1 File — (DOCX) [file pone.0351752.s004.docx]

**Table S1.** Reference strain sequences of *Colletotrichum* obtained from GenBank and used in phylogenetic analyses. Letter “T” identifies type material.

| **Species** | **Isolate ID** | **GAPDH** | ***tub2*** | ***MAT1-2*** |
| --- | --- | --- | --- | --- |
| *Colletotrichum queenslandicum* | ICMP 18705 | JX010036 | JX010412 |  |
| *C. musae* | ICMP 17817 | JX010015 | JX010395 |  |
| *C. siamense* | CPO 27.601 | MN737352 | MN848369 | MN854418 |
| *C. aeschynomenes* | ICMP 17673 | OR475941 | OR050783 | OQ198537 |
| *C. queenslandicum* | ICMP 1778 | PP948701 | PP952768 | PP622748 |
| *G. cingulata f. sp. camelliae* | ICMP 10646 | JX009993 | JX010437 |  |
| *C. perseae* | GA335 | KX620259 | KX620358 | KX620192 |
| *C. aenigma* | ICMP 18686 | JX009913 | JX010390 |  |
| *C. alienum* | ICMP 12071 | PP506777 | PP506923 | MG584592 |
| *C. jiangxiense* | CGMCC 3.17363 | PQ653933 | PQ566709 | PQ653927 |
| *C. tropicale* | CBS 124949 | PP510211 | PP510213 | OQ791437 |
| *C. perseae* | GA272 | KX620255 | KX620354 | KX620188 |
| *C. chrysophilum* | CPO 27.709 | MN737344 | MN848365 | MN854412 |
| *C. perseae* | GA320 | KX620257 | KX620356 | KX620190 |
| *C. musae* | ICMP 19119 | PQ664489 | PQ187596 | DQ002817.2 |
| *C. rhexiae* | CBS 133134 | MZ664046 | JX145179 | JX145290 |
| *C. henanense* | CGMCC 3.17354 | PP849819 | PP870989 | KM610175 |
| *C. theobromicola* | ICMP 18649 | OR475996 | PP455358 | ON532816 |
| *C. grevilleae* | CBS 132879 | KC297010 | KC297102 |  |
| *C. wuxiense* | CGMCC 3.17894 | KU252045 | KU252200 | KU251722 |
| *C. tropicale* | CPO 27.719 | MN737355 | MN848370 | MN854420 |
| *C. siamense* | ICMP 17795 | JX010051 | JX010393 |  |
| *C. clidemiae* | ICMP 18658 | JX009989 |  |  |
| *C. cigarro* | CPO 27.939 | MN737349 |  | MN854415 |
| *C. tropicale* | CPO 27.477 | MN737354 |  | MN854419 |
| *C. temperatum* | CBS 133122 | MZ664045 |  |  |
| *C. xanthorrhoeae* | ICMP 17903 | GU174563 |  |  |
| *C. aotearoa* | ICMP 18537 | JX010005 | JX010420 | KC888930 |
| *C. siamense* | CPO 27 415 | MN737351 |  | MN854417 |
| *C. nupharicola* | ICMP 17938 | JX009936 | JX010397 |  |
| *C. gloeosporioides* | LF318 | KJ954828 | KJ955275 | KJ954541 |
| *C. tropicale* | CPO 27.830 | MN737356 | MN848371 | MN854421 |
| *C. alatae* | ICMP 17919 | JX009990 |  |  |
| *C. hebeiense* | MFLUCC 13-0726 | KF377495 | KF288975 |  |
| *C. proteae* | CBS 132882 | KC297009 | KC297101 |  |
| *C. changpingense* | MFLUCC 15-0022 | MZ664048 | MZ673952 |  |
| *C. siamense* | ICMP 18578 | JX009924 | JX010404 | JQ899289 |
| *C. nupharicola* | ICMP 18187 | JX009972 | JX010398 | JX145319 |
| *C. fructicola* | ICMP 18646 | JX010032 | JX010409 |  |
| *C. horii* | ICMP 17968 | JX009939 | JX010378 |  |
| *C. psidii* | ICMP 19120 | JX009967 | JX010443 | KC888931 |
| *C. asianum* | ICMP 18580 | JX010053 | JX010406 |  |
| *C. grossum* | CGMCC 3.17614 | PQ505472 | PQ505473 |  |
| *C. viniferum* | GZAAS 5.08601 | JN412798 | JN412813 |  |
| *C. asianum* | GM595 | JQ894623 | JQ894601 | JQ894554 |
| *C. horii* | ICMP 10492 | JX009964 | JX010450 | JQ807840 |
| *C. gloeosporioides* | ICMP 17821 | JX010056 | JX010445 |  |
| *C. kahawae subsp. ciggaro* | ICMP 17922 | JX010042 | JX010432 |  |
| *C. perseae* | GA319 | KX620256 | KX620355 | KX620189 |
| *C. chrysophilum* | CPO 27.408 | MN737343 | MN848364 | Not Available |
| *C. salsolae* | ICMP 19051 | JX009916 | JX010403 | KC888925 |
| *C. aenigma* | ICMP 18608 | JX010044 | JX010389 | KM360143 |
| *C. conoides* | CGMCC 3.17615 | KP890162 | KP890174 |  |
| *C. siamense* | CPO 27 400 | MN737350 |  | MN854416 |
| *C. chrysophilum* | CMM 4268 | KX094183 | KX094285 | KX094325 |
| *C. fructicola* | ICMP 18581 | JX010033 | JX010405 |  |
| *C. ti* | ICMP 4832 | JX009952 | JX010442 | KM360146 |
| *C. fructivorum* | CBS 133125 |  | JX145196 | JX145300 |
| *C. perseae* | GA100 | PQ178840 | PQ195571 | PQ559326 |
| *C. cordylinicola* | ICMP 18579 | JX009975 | JX010440 |  |

**Table S2.** Reference strain sequences of Botryosphaeriaceae obtained from GenBank and used in phylogenetic analyses. Letter “T” identifies type material.

| **Species** | **Isolate ID** | **ITS** | ***tef1-α*** | ***tub2*** |  |
| --- | --- | --- | --- | --- | --- |
| *B. dothidea* | CBS 115476 = CMW 8000**T** | AY236949 | AY236898 | AY236927 | |
| *B. dothidea* | CBS 110302 = CAP 007 | AY259092 | AY573218 | EU673106 | |
| *Neofusicoccum arbuti* | CBS 116131 = AR 4014**T** | AY819720 | KF531792 | KF531793 | |
| *N. arbuti* | CBS 117090 = UW13 | AY819724 | KF531791 | KF531794 | |
| *N. australe* | CBS 139662 = CMW 6837**T** | AY339262 | AY339270 | AY339254 | |
| *N. australe* | CMW 6853 | AY339263 | AY339271 | AY339255 | |
| *N. brasiliense* | CMM 1285 | JX513628 | JX513608 | KC794030 | |
| *N. brasiliense* | CMM 1338**T** | JX513630 | JX513610 | KC794031 | |
| *N. cordaticola* | CBS 123634 = CMW 13992**T** | EU821898 | EU821868 | EU821838 | |
| *N. cordaticola* | CBS 123635 | EU821903 | EU821873 | EU821843 | |
| *N. cryptoaustrale* | CBS 122813 = CMW 23785**T** | FJ752742 | FJ752713 | FJ752756 | |
| *N. dianense* | CSF6075 = CGMCC3.20082**T** | MT028605 | MT028771 | MT028937 | |
| *N. eucalypticola* | CBS 115679 = CMW 6539**T** | AY615141 | AY615133 | AY615125 | |
| *N. eucalypticola* | CBS 115766 = CMW 6217 | AY615143 | AY615135 | AY615127 | |
| *N. eucalyptorum* | CBS 115791 = CMW 10125 = BOT 24**T** | AF283686 | AY236891 | AY236920 | |
| *N. eucalyptorum* | CBS 145975 = CPC 29337 | MT587477 | MT592190 | MT592682 | |
| *N. hellenicum* | CERC 1947 = CFCC 50067**T** | KP217053 | KP217061 | KP217069 | |
| *N. hellenicum* | CERC 1948=CFCC 50068 | KP217054 | KP217062 | KP217070 | |
| *N. hongkongense* | CERC2973 = CGMCC3.18749**T** | KX278052 | KX278157 | KX278261 | |
| *N. hongkongense* | CERC 2968 = CGMCC 3.18748 | KX278051 | KX278156 | KX278260 | |
| *N. kwambonambiense* | CBS 123639 = CMW 14023**T** | EU821900 | EU821870 | EU821840 | |
| *N. kwambonambiense* | CBS 123641 = CMW 14140 | EU821919 | EU821889 | EU821859 | |
| *N. lumnitzerae* | CBS 139674 = CMW 41469**T** | KP860881 | KP860724 | KP860801 | |
| *N. lumnitzerae* | CBS 139675 = CMW 41228 | MT587480 | MT592193 | MT592685 | |
| *N. luteum* | CBS 110497 = CPC 4594 = CAP 037 | EU673311 | EU673277 | EU673092 | |
| *N. luteum* | CBS 110299 = LM 926 = CAP 002**T** | AY259091 | KX464688 | DQ458848 | |
| *N. macroclavatum* | CBS 118223 = CMW 15955 = WAC 12444**T** | DQ093196 | DQ093217 | DQ093206 | |
| *N. magniconidium* | CSF5876 = CGMCC3.20077**T** | MT028612 | MT028778 | MT028944 | |
| *N. mangiferae* | CBS 118531 = CMW 7024**T** | AY615185 | DQ093221 | AY615173 | |
| *N. mediterraneum* | CBS 121558 | GU799463 | GU799462 | GU799461 | |
| *N. mediterraneum* | CBS 121718 = CPC 13137**T** | GU251176 | GU251308 | GU251836 | |
| *N. microconidium* | CERC3497 = CGMCC3.18750**T** | KX278053 | KX278158 | KX278262 | |
| *N. microconidium* | CBS 118821 = CMW 13998 | MT587497 | MT592212 | MT592704 | |
| *N. ningerense* | CSF6028 = CGMCC3.20078T | MT028613 | MT028779 | MT028945 | |
| *N. nonquaesitum* | CBS 126655 = L3IE1 = PD484**T** | GU251163 | GU251295 | GU251823 | |
| *N. nonquaesitum* | CBS 133501 = UCR532 | MT587498 | MT592213 | MT592705 | |
| *N. occulatum* | CBS 128008 = MUCC 227**T** | EU301030 | EU339509 | EU339472 | |
| *N. occulatum* | MUCC 286 = WAC 12395 | EU736947 | EU339511 | EU339474 | |
| *N. parvum* | CBS 138823 = ICMP 8003 = CMW 9081**T** | AY236943 | AY236888 | AY236917 | |
| *N. parvum* | CBS 110301 = CAP 074 | AY259098 | AY573221 | EU673095 | |
| *N. parviconidium* | CSF5667 = CGMCC3.20074**T** | MT028615 | MT028781 | MT028947 | |
| *N. pennatisporum* | WAC 13153 = MUCC 510**T** | EF591925 | EF591976 | EF591959 | |
| *N. pistaciae* | CBS 595.76**T** | KX464163 | KX464676 | KX464953 | |
| *N. podocarpi* | CBS 131677 = CMW 35494 | MT587508 | MT592223 | MT592715 | |
| *N. podocarpi* | CBS 131678 = CMW 35499 | MT587509 | MT592224 | MT592716 | |
| *N. protearum* | CBS 114176 = CPC 1775 = JT 189**T** | AF452539 | KX464720 | KX465006 | |
| *N. protearum* | CBS 115177 = CPC 4357 | FJ150703 | MT592239 | MT592731 | |
| *N. ribis* | CBS 115475 = CMW 7772**T** | AY236935 | AY236877 | AY236906 | |
| *N. ribis* | CBS 124923 = CMW 28320 | FJ900608 | FJ900654 | FJ900635 | |
| *N. ribis* | CBS 124924**T** | FJ900607 | FJ900653 | FJ900634 | |
| *N. ribis* | CBS 123645 = CMW 14058**T** | EU821904 | EU821874 | EU821844 | |
| *N. ribis* | CBS 123646 = CMW 14060 | EU821905 | EU821875 | EU821845 | |
| *N. sinense* | CGMCC3.18315**T** | KY350148 | KY817755 | KY350154 | |
| *N. sinoeucalypti* | CERC2005 = CGMCC3.18752**T** | KX278061 | KX278166 | KX278270 | |
| *N. sinoeucalypti* | CERC3415 | KX278063 | KX278168 | KX278272 | |
| *N. stellenboschiana* | CBS 110864 = CPC 4598 | AY343407 | AY343348 | KX465047 | |
| *N. terminaliae* | CBS 125263 = CMW 26679**T** | GQ471802 | GQ471780 | KX465052 | |
| *N. terminaliae* | CBS 125264 = CMW 26683 | GQ471804 | GQ471782 | KX465053 | |
| *N. ursorum* | CBS 122811 = CMW 24480**T** | FJ752746 | FJ752709 | KX465056 | |
| *N. ursorum* | CBS 122812 = CMW 23790 | FJ752745 | FJ752708 | KX465057 | |
| *N. yunnanense* | CSF6142 = CGMCC3.20083**T** | MT028667 | MT028833 | MT028999 | |
| *N. viticlavatum* | CBS 112878 = CPC 5044 = JM 86**T** | AY343381 | AY343342 | KX465058 | |
| *N. viticlavatum* | CBS 112977 = STE-U 5041 | AY343380 | AY343341 | KX465059 | |
| *N. vitifusiforme* | CBS 110887 = CPC 5252 = JM5**T** | AY343383 | AY343343 | KX465061 | |
| *N. vitifusiforme* | CBS 121112 = STE-U 5912 | EF445349 | EF445391 | KX465016 | |

**Table S3.** Reference strain sequences of *Diaporthe* obtained from GenBank and used in phylogenetic analyses. Letter “T” identifies type material.

| **Species** | **Isolate ID** | **ITS** | ***tef1-α*** | ***tub2*** |
| --- | --- | --- | --- | --- |
| *Cytospora disciforme* | CBS 116827 T | KY051801 | KX965072 | KX964907 |
| *Diaporthe anacardii* | CBS 720.97 T | KC343024 | KC343750 | KC343992 |
| *D. baccae* | CBS 136972 T | KJ160565 | KJ160597 |  |
| *D. baccae* | CBS 136971 T | KJ160564 | KJ160596 |  |
| *D. baccae* | CPC 20586 | KJ160568 | KJ160600 |  |
| *D. baccae* | CPC 20583 | KJ160566 | KJ160598 |  |
| *D. baccae* | CPC 29659 | MG281006 | MG281527 | MG281179 |
| *D. baccae* | CPC 20585 | KJ160564 | KJ160596 |  |
| *D. camelliae-sinensis* | SAUCC194.92 T | MT822620 | MT855932 | MT855817 |
| *D. camelliae-sinensis* | SAUCC194.103 | MT822631 | MT855943 | MT855828 |
| *D. canthii* | CBS 132533 T | JX069864 | KC843120 | KC843230 |
| *D. chamaeropis* | CBS 454.81 T | KC343048 | KC343774 | KC344016 |
| *D. chamaeropis* | CBS 753.70 | KC343049 | KC343775 | KC344017 |
| *D. cissampeli* | CPC 2732 T | KX228273 |  | KX228384 |
| *D. cytosporella* | FAU461 T | KC843307 | KC843116 | KC843221 |
| *D. elaeagni-glabrae* | LC4802 | KX986779 | KX999171 | KX999212 |
| *D. elaeagni-glabrae* | LC4806 | KX986780 | KX999172 | KX999213 |
| *D. foeniculina* | AR5142 | KC843301 | KC843110 | KC843215 |
| *D. foeniculina* | MEP 12891 | KC843305 | KC843114 | KC843219 |
| *D. foeniculina* | CBS 187.27 | MH854926 |  |  |
| *D. foeniculina* | DP0392 | KC843296 | KC843105 | KC843210 |
| *D. foeniculina* | AR5144 | KC843302 | KC843111 | KC843216 |
| *D. foeniculina* | DP0391 | KC843295 | KC843104 | KC843209 |
| *D. foeniculina* | DP0454 | KC843297 | KC843106 | KC843211 |
| *D. foeniculina* | AR5145 | KC843306 | KC843115 | KC843220 |
| *D. foeniculina* | AR5143 | KC843294 | KC843103 | KC843208 |
| *D. foeniculina* | CBS 123208 T | KC343104 | KC343830 | KC344072 |
| *D. forlicesenica* | MFLUCC 17-1015 T | KY964215 | KY964171 | KY964099 |
| *D. hickoriae* | CBS 145.26 T | KC343118 | KC343844 | KC344086 |
| *D. inconspicua* | CBS 133813 T | KC343123 | KC343849 | KC344091 |
| *D. inconspicua* | URM7776 | MG696772 | MG710414 | MG710395 |
| *D. isoberliniae* | CPC 22549 T | KJ869133 |  | KJ869245 |
| *D. lutescens* | SAUCC194.36 T | MT822564 | MT855877 | MT855761 |
| *D. macintoshii* | BRIP 55064a T | KJ197289 | KJ197251 | KJ197269 |
| *D. maytenicola* | CPC 21896 T | KF777157 |  | KF777250 |
| *D. melastomatis* | SAUCC194.55 T | MT822583 | MT855896 | MT855780 |
| *D. melastomatis* | SAUCC194.80 | MT822608 | MT855920 | MT855805 |
| *D. nebulae* | PMM1681 T | KY511337 | MH708552 | KY511369 |
| *D. nebulae* | Phom240 | KY511315 | MH708543 | KY511346 |
| *D. oncostoma* | CBS 589.78 | KC343162 | KC343888 | KC344130 |
| *D. oncostoma* | CBS 100454 | KC343160 | KC343886 | KC344128 |
| *D. parapterocarpi* | CBS 137986 T | KJ869138 |  | KJ869248 |
| *D. parvae* | PSCG 034 | MK626919 | MK654858 | MK691248 |
| *D. parvae* | PSCG035 | MK626920 | MK654859 | MK691249 |
| *D. phillipsii* | CAA817 T | MK792305 | MK828076 | MN000351 |
| *D. phillipsii* | CAA818 | MK792307 | MK828078 | MN000352 |
| *D. poincianellae* | URM 7932 T | MH989509 | MH989538 | MH989537 |
| *D. portugallica* | CPC 34247 T | MH063905 | MH063911 | MH063917 |
| *D. portugallica* | CPC 34248 | MH063906 | MH063912 | MH063918 |
| *D. pseudoinconspicua* | E13 | MH122535 | MH122530 | MH122521 |
| *D. pseudoinconspicua* | G26 | MH122538 | MH122533 | MH122524 |
| *D. pterocarpi* | MFLUCC 10-0571 T | JQ619899 | JX275416 | JX275460 |
| *D. pterocarpi* | MFLUCC 10-0588 | JQ619900 | JX275417 | JX275461 |
| *D. pungensis* | SAUCC194.112 T | MT822640 | MT855952 | MT855837 |
| *D. pungensis* | SAUCC194.89 | MT822617 | MT855929 | MT855814 |
| *D. ravennica* | MFLUCC 15-0479 T | KU900335 | KX365197 | KX432254 |
| *D. ravennica* | MFLUCC 17-1029 | KY964191 | KY964147 | KY964075 |
| *D. saccarata* | CBS 116311 T | KC343190 | KC343916 | KC344158 |
| *D. stictica* | CBS 370.54 T | KC343212 | KC343938 | KC344180 |
| *D. vangueriae* | CBS 137985 T | KJ869137 |  | KJ869247 |
| *D. velutina* | LC4421 | KX986790 | KX999182 | KX999223 |
| *D. velutina* | LC4419 | KX986789 | KX999181 | KX999222 |
| *D. zaobaisu* | CGMCC 3.19598 T | MK626922 | MK654855 | MK691245 |
| *D. zaobaisu* | PSCG032 | MK626923 | MK654856 | MK691246 |

**Table S4.** Table S4. Monthly total precipitation recorded at the CIMIS Irvine station (Station 75), California, from October 2020 through August 2023.

| **Year** | **Month** | **Total Precipitation (mm)** |
| --- | --- | --- |
| 2020 | October (10) | 1.4 |
|  | November (11) | 15.4 |
|  | December (12) | 22.8 |
| 2021 | January (1) | 76.8 |
|  | February (2) | 1.3 |
|  | March (3) | 37.7 |
|  | April (4) | 1.6 |
|  | May (5) | 1.8 |
|  | June (6) | 0.3 |
|  | July (7) | 1.6 |
|  | August (8) | 4.8 |
|  | September (9) | 2.8 |
|  | October (10) | 20.6 |
|  | November (11) | 5.9 |
|  | December (12) | 147.4 |
| 2022 | January (1) | 3.1 |
|  | February (2) | 9.4 |
|  | March (3) | 30.1 |
|  | April (4) | 5.4 |
|  | May (5) | 0.6 |
|  | June (6) | 5.6 |
|  | July (7) | 7.2 |
|  | August (8) | 1.4 |
|  | September (9) | 7.3 |
|  | October (10) | 12.1 |
|  | November (11) | 48.5 |
|  | December (12) | 84.3 |
| 2023 | January (1) | 152.9 |
|  | February (2) | 94 |
|  | March (3) | 154.8 |
|  | April (4) | 1.3 |
|  | May (5) | 27.2 |
|  | June (6) | 5 |
|  | July (7) | 0.2 |
|  | August (8) | 12.7 |
