# Supplementary material for: Influence of fruit maturity and variety on seasonal postharvest fungal diseases and vascular streaking in California Avocados
Source: PLoS One. 2026 Jun 25;21(6):e0351752. doi: 10.1371/journal.pone.0351752 (PMC13298749; doi:10.1371/journal.pone.0351752)
Supplement: Attachment — Submitted filename: Response to reviewers R1 final.docx [file pone.0351752.s007.docx]

**Response to Editorial comments #1**

Dear Editor,

Please find the detailed responses below and the corresponding revisions and corrections in the re-submitted files.

**Comment 1**: Please ensure that your manuscript meets PLOS ONE's style requirements, including those for file naming.

**Response 1**: The manuscript body, title, authors, and affiliation formats were checked. The current address for author C. M. Asensio was added as follows (Page 1, lines 14-15):

“# a Department of Food Science and Human Nutrition, University of Illinois Urbana-Champaign, Urbana, IL 618101, U.S.A.”

**Comment 2:** Please note that PLOS One has specific guidelines on code sharing for submissions in which author-generated code underpins the findings in the manuscript. In these cases, we expect all author-generated code to be made available without restrictions upon publication of the work.

**Response 2:** According to the journal guidelines, a repository for the codes used in this study was generated. The code used to generate the results in this study is publicly available at Zenodo: <https://zenodo.org/records/20218289> .

**Comment 3:** Thank you for stating the following financial disclosure: “Eurosemillas, S.A. (awarded to M.L.A.).” Please state what role the funders took in the study. If the funders had no role, please state: "The funders had no role in study design, data collection and analysis, decision to publish, or preparation of the manuscript."

**Response 3**: Additional information was incorporated into the financial disclosure as suggested. “Funding: Eurosemillas, S.A. (awarded to M.L.A.). The funders had no role in study design, data collection and analysis, decision to publish, or preparation of the manuscript.”

**Comment 4.** Please note that funding information should not appear in any section or other areas of your manuscript. We will only publish funding information present in the Funding Statement section of the online submission form. Please remove any funding-related text from the manuscript.

**Response 4:** Thanks, the financial disclosure statement was removed from the manuscript as suggested.

**Comment 5:** In the online submission form, you indicate that your data is not available for proprietary reasons and have provided a contact point for accessing this data. Please note that your current contact point is a co-author on this manuscript. According to our Data Policy, the contact point must not be an author on the manuscript and must be an institutional contact, ideally not an individual. Please revise your data statement to a non-author institutional point of contact, such as a data access or ethics committee, and send this to us via return email. Please also include contact information for the third party organization, and please include the full citation of where the data can be found.

**Response 5:** Thank you for your clarifications regarding the data availability requirements. We have deposited the dataset and associated R scripts in the Zenodo repository and obtained a DOI for the record. The repository includes all data and code necessary to reproduce the analyses reported in the manuscript. At this stage, the files are under a temporary embargo during the peer-review process. However, we confirm that all data and code will be made fully publicly available without restriction upon publication, in full compliance with PLOS ONE’s Data Availability policy.

The following information was included in our Data Availability Statement:

“DNA sequence data for all fungal isolates analyzed in this study have been deposited in GenBank, and accession numbers are provided in the manuscript and Supporting Information. We are currently sharing data specific to our analyses so that they can be legally distributed. All data and code supporting the findings will be publicly available upon publication of the manuscript at Zenodo: <https://zenodo.org/records/20313537>.

**Comment 6:** Please include your full ethics statement in the ‘Methods’ section of your manuscript file. In your statement, please include the full name of the IRB or ethics committee who approved or waived your study, as well as whether or not you obtained informed written or verbal consent. If consent was waived for your study, please include this information in your statement as well.

**Comment 6:** Thank you for your comment. We would like to clarify that this study did not involve human participants or vertebrate animals. The research was conducted on plant materials (avocado) and associated pathogens. Therefore, Institutional Review Board (IRB) approval and informed consent are not applicable to this research.

The following information is included in the Ethics Statement, because the study involved “Field Research”:

“Field sampling of avocado fruit was conducted in a University of California–managed research station in California. Permission to collect fruit and plant material was granted by the University of California, Riverside Agricultural Operations. No government permits were required for this work.”

**Comment 7:** We note that you have referenced (DM pattern observed in our study and previous unpublished data.) which has currently not yet been accepted for publication. Please remove this from your References and amend this to state in the body of your manuscript: (ie “Bewick et al. [Unpublished]”) as detailed online in our guide for authors

**Response 7:** Thank you for your comment. We have modified this to meet the journal guidelines. Unpublished data were cited in the manuscript as “Camiletti et al. [Unpublished]” (page 25, lines 485-486).

**Comment 8**: We note that Supporting Figures 1, 2, and 3 in your submission contain copyrighted images. All PLOS content is published under the Creative Commons Attribution License (CC BY 4.0), which means that the manuscript, images, and Supporting Information files will be freely available online, and any third party is permitted to access, download, copy, distribute, and use these materials in any way, even commercially, with proper attribution. For more information, see our copyright guidelines: http://journals.plos.org/plosone/s/licenses-and-copyright.

We require you to either (1) present written permission from the copyright holder to publish these figures specifically under the CC BY 4.0 license, or (2) remove the figures from your submission:

**Response 8:** Thank you for your comment regarding Supporting Figures 1–3. We would like to clarify that the figures in question are original and author-generated, and the copyright is held by the first author of the manuscript. These images have not been reproduced from any previously published source and do not include third-party copyrighted material. As such, no additional copyright permission is required. We confirm that these figures can be published under the Creative Commons Attribution (CC BY 4.0) license in accordance with PLOS ONE’s policies. Please let us know if any further clarification is needed.

**Comment 9**: If the reviewer comments include a recommendation to cite specific previously published works, please review and evaluate these publications to determine whether they are relevant and should be cited. There is no requirement to cite these works unless the editor has indicated otherwise.

**Response 9:** We appreciate the clarification. We were suggested to include a citation in our literature. That change is reflected in the manuscript

**Comment 10:** Please review your reference list to ensure that it is complete and correct. If you have cited papers that have been retracted, please include the rationale for doing so in the manuscript text, or remove these references and replace them with relevant current references. Any changes to the reference list should be mentioned in the rebuttal letter that accompanies your revised manuscript. If you need to cite a retracted article, indicate the article’s retracted status in the References list and also include a citation and full reference for the retraction notice.

**Response 10:** Thank you for your comment. /

**Response to Editorial comments #2**

Please find the detailed response below and the corresponding revision and corrections in the re-submitted files.

**Comment 1:** We've checked your submission and before we can proceed, we need you to address the following issues:

1. Thank you for your detailed and excellent response. Original images generated by the authors are fully acceptable. To ensure that the information is made known to all future readers and editors, please update your captions S1 Fig., S2 Fig., and S3 Fig. in your manuscript to note that the images were generated or captured by your research team. Once this is complete your paper will be fine to move forward in our system.

**Response 1:** Thank you for your positive assessment. We have revised the captions of Supporting Information figures S1–S3 to explicitly state that all images were generated and captured by our research team. The updated captions are as follows:

S1 Fig. Lesions caused by N. nonquaesitum on ‘Hass’ (A), I49LB (B), and I55B (C) fruits. Images generated and captured by the authors.

S2 Fig. Lesions caused by C. perseae on ‘Hass’ (A), I49LB (B), and ‘Luna UCR’ (C) fruits. Images generated and captured by the authors.

S3 Fig. Lesions caused by D. foeniculina on ‘Hass’ (A), I49LB (B), and I55B (C) fruits. Images generated and captured by the authors**.**

**Response to Review #1**

Dear reviewer,

Thank you for your insightful comments. Please find the detailed responses below and the corresponding revisions and corrections in the re-submitted files.

**Comment 1:** The research work is mainly descriptive, evaluating the responses of different genetic backgrounds to three main avocado postharvest diseases. The experimental design, methodological approach, and implementation are sound, and the presentation of the results is clear. Further elaboration of the discussion part is suggested.

**Response 1:** We appreciate the positive feedback and revised the Discussion to further elaborate on key findings and study limitations (lines 631-635; 658-660; 665-668; 671-673).

**Comment 2:** There is presentation of a considerable results’ dataset, the analysis of which shapes indicative cultivar profiles regarding harvest time and windows, main disease resistance performance, and in relation to DM which function as (the) fruit quality indicator

**Response 2:** We thank the reviewer for recognizing the value of the dataset and the relevance of the cultivar-specific analyses linking harvest timing, disease response, and dry matter accumulation.

**Comment 3:** Though there are scarce references to molecules (persin) or genetic background (Haas or non- Haas -related) factors that could reason, connect or support phenotypic responses, there is no further research (molecular/genomics) on these factors at the present time.

**Response 3:** We appreciate this insightful observation and agree that further molecular and genomic studies would be valuable to better explain the phenotypic responses observed.

**Comment 4:** On the contrary, the molecular (phylogenetic) characterization of the fungal pathogen isolates via multilocus analysis using selected markers per pathogen goes in depth and covers the needs even for “tougher” species complexes like Colletotrichum spp (use of the most informative markers).

**Response 4:** We thank the reviewer for acknowledging the depth of the multilocus phylogenetic analyses and the use of informative molecular markers for pathogen characterization.

**Comment 5:** It would be useful to report on the fertilization (soil nutrient analyses if available as well) and plant protection regimes followed during the three different seasons. Was there a horizontal regime applied to all cultivars per season or adapted to each cultivar’s specific needs? Was this (either the former or later case) adapted to each season with regards to the climatic conditions of each season? Please evaluate if it would be informative to comment on and also discuss these aspects (fertilization/plant protection followed) with regards to cultivars’ responses to the pathogens (e.g. if there is relevant data available from previous research, or even if these were parameters that you would like to study in the near future).

**Response 5:** We thank the reviewer for this valuable suggestion. We added information on fertilization and plant protection practices to the Materials and Methods section (lines 161-174) and briefly expanded the Discussion (lines 665-668) to clarify that all cultivars were maintained under the same standard minimum fertilization program, with no fungicide or insecticide applications.

**Comment 6:** What I am missing is a conclusive (or suggestion-type) graph or table gathering the information of individual results (DM, Severity scores, putatively Lesion severities as well...) presented in the individual figures and tables. Since these datasets/results are not fed yet in a predictive (DSStype) model along with other geoclimatic and field-management actions to suggest harvest time(s)/windows, it would probably be interesting to present a preliminary graph or table using e.g. a selected DM value(s) filter and present collectively harvest windows, pathogen/disease risk, responses, etc. for each cultivar. Please evaluate if this could embrace all your results in a collective format that could further lighten your work and based on the graph/table give you the opportunity to discuss and suggest a putative roadmap for cultivars’ best use.

**Response 6:** We thank the reviewer for this helpful suggestion. We added a new summary table (Table 5) integrating seasonal dry matter accumulation patterns, observed disease trends, and preliminary harvest considerations for each cultivar and selection (lines 726-728).

**Comment 7:** 1. For the susceptibility tests there is selection of certain species. Please comment on the basis of your choice (e.g. most prevalent/most prevalent in general/bibliography based, etc.) to support the choice of certain and not all different species identified.

**Response 7:** We thank the reviewer for this comment. We added clarification to the Materials and Methods (lines 331-332) indicating that the pathogens selected for susceptibility assays corresponded to the most prevalent species identified during the surveys.

**Comment 8:** For susceptibility tests only one isolate (lines 271-272) per species is used. Please evaluate/comment on the probability that cultivar responses may vary in case a second or third isolate from the same species is used.

**Response 8:** We thank the reviewer for this important observation. We added a sentence to the Discussion acknowledging this limitation and noting that future studies with multiple isolates per species would help evaluate potential intraspecific variability in aggressiveness and cultivar responses (lines 671-673).

**Comment 9:** Line 217; please check if reference of Table 1 is correct.

**Response 9:** The incorrect table reference was removed.

**Comment 10:** 4. Line 407; typo “XXXXX”

**Response 10:** We thank the reviewer for noting this. The placeholder was corrected and the missing information was added (line 496).

**Comment 11:** Lines 466-467; typo single/plural

**Response 11:** We thank the reviewer for noting this. The sentence was corrected (line 568).

**Comment 12:** Line 560; does this (most frequent pathogens) refer to the diseases “respectively” or is mentioned in a generalized way?

**Response 12:** This was intended in a general sense, as pathogen predominance can vary by disease.

**Comment 13:** Line 599-600; Does “fruit maturity” refer to DM-related concept or in a generalized way?

**Response 13:** We revised the sentence to clarify that this refers to DM-based maturity (lines 721-722).

**Comment 14:** In Figure 5 “Others” is a notable share of the Relevant Frequency. Could you report (in the manuscript) the most prevalent genera? Please evaluate if this can be also connected to your comment for endophytes (Lines 520-522), and elaborate/discuss if you think it is relevant.

**Response 14:** We thank the reviewer for this valuable suggestion. We clarified the main genera included in “Others” and added a sentence to the Discussion (lines 631-635) noting that some of these fungi may occur as opportunistic endophytes or secondary colonizers, although their specific role in postharvest disease development was beyond the scope of this study.

**Response to Review #2**

Dear reviewer,

Thank you for your insightful comments. Please find the detailed responses below and the corresponding revisions and corrections in the re-submitted files. We accepted most of the minor comments throughout the text; you can see them in the track-changed version.

**General Comment:** This manuscript is good for learning about avocado varieties and their yields, as well as identifying the fungal diseases that affect them. The research is also good because a large number of fungi have been identified.
**Response:** We thank you for the very positive and thoughtful comment.

**Comment 1:** Suggested to change title to “Fruit Maturity and Variety's Seasonal Effects on Avocado Postharvest Fungal Diseases and Disorders in California”.

**Response 1:** We thank the reviewer for the suggestion. We modified the current title according to your and other reviewers' suggestions.

**Comment 2:** In the abstract “You must mention the aims of study , the variety of avocados , mention the primers and then the results”.

**Response 2:** We thank the reviewer for this suggestion. The first part of the abstract was revised to include the study aims, avocado varieties, molecular regions used, and main results (lines 23-28).

**Comment 3:** Introduction , reviewer suggested deleting “Consequently, the dry matter percentage (DM) has been developed as an indicator of maturity. In California, minimum maturity standards are based on DM thresholds established to ensure acceptable fruit quality. For example, the minimum DM standard for ‘Hass’ is 20.8% [26]. These thresholds are largely derived from studies by Lee et al. and Ranney et al., which demonstrated strong correlations between dry matter content and sensory quality attributes such as flavor and texture [27,28] . While minimum maturity standards have been established for many avocado varieties, they remain undefined for numerous others. “

**Response 3:** We agree that this section was lengthy, but we consider the information important because DM is discussed throughout the manuscript. The paragraph was revised to make it more concise (lines 105-109).

**Comment 4:** “Number of orchards or locations must be determined, the age of trees ,Rootstocks and Soil type”

**Response 4:** We thank the reviewer for this comment. This information was added to the “Avocado fruit collection and dry matter determination” section (lines 159-165).

**Comment 5:** You must determine the maturity of fruits

**Response 5:** Fruit maturity was determined based on harvest timing within the expected seasonal harvest window of each variety and by measuring fruit dry matter percentage (DM). Because established minimum DM maturity standards are currently available only for ‘Hass’ and ‘GEM’, maturity stages for the remaining varieties and unreleased selections were inferred relative to their seasonal harvest progression (lines 165-168).

**Comment 6:** Three boxes per replicate then three boxes per variety

**Response 6:** We thank the reviewer for noting this unclear wording. This section was revised to clarify the number of boxes and their assignment to storage periods (194-197).

**Comment 7:** Number of fruits evaluated per treatment

**Response 7:** We clarified that all 25 fruits in each carton were evaluated (line 209).

**Comment 8:** Must be mentioned A formula for Disease Severity Index (DSI)

**Response 8:** We did not calculate a DSI. Disease severity scores were analyzed as ordinal variables, as described in the Statistical analysis section.

**Comment 9:** How many plates per replicate?

**Response 9:** Only symptomatic fruits were used for fungal isolation; therefore, there was no predetermined number of plates per replicate.

**Comment 10:** Table 3, “italic”

**Response 10:** Botryosphaeriaceae is a family name and was therefore not italicized. This formatting was kept consistently throughout the manuscript.

**Comment 11:** reviewer left a “?” for this sentence: For instance, ‘GEM’ fruits are more susceptible to fungal pathogens in the earliest harvest, as expected given their low dry matter (DM), but this does not translate into a higher frequency of observed rots.

**Response 11:** We thank the reviewer for pointing out that this sentence was unclear. The sentence was revised to clarify the intended meaning (658-660).

**Comment 12:** “this reference is old”

**Response 12:** We agree that some references are older; however, we retained this citation because it represents the original source of the finding.

**Response to Review #3**

Dear reviewer,

Thank you for your insightful comments. Please find the detailed responses below and the corresponding revisions and corrections in the re-submitted files.

**Comment 1:** The manuscript reports studies on the etiology of postharvest fruit rots of avocado in California and the influence of avocado cultivar (some commercial cultivars and breeding selections), fruit maturity (as % of dry matter, DM), and cold storage period on disease severity (score 0-3 for stem-end rots and 0-5 for body rots) on fruits harvested monthly for 3 consecutive seasons. The influence on vascular streaking was also addressed. Susceptibility tests were also conducted with fruits artificially inoculated with three selected pathogenic fungal species.
In my opinion, this research is of very high quality. The subject is of great interest, the volume of work is considerable, and the information is new and valuable because gives some keys to the avocado production sector to maximize profit by minimizing postharvest disease incidence. The manuscript is very well written, clear and well organized in subsections. In general, the experiments are well designed and repeated sufficiently, proper statistical analyses were performed, the results are adequately presented in tables and figures, and the data support the conclusions of the authors. The introduction and discussion are complete, and the number and quality of the literature references are appropriate.

I am only wondering why a correlation analysis (Spearman??) between DM and disease severity was not performed for each cultivar, harvest date, and perhaps the first storage period. It seems, reading the abstract (L28), that such analysis could be expected as an outcome from the study.
Since I am not familiar with work at the molecular level, I cannot comment on this part of the research, and I recommend the editor to invite additional expert reviewers to address the molecular identification of fungal species and phylogenetic analyses.
**Response 1:** We sincerely thank the reviewer for the very positive and thoughtful evaluation of our manuscript, and for highlighting the potential interest of examining correlations between dry matter (DM) and disease severity. We agree that correlation analyses (e.g., Spearman correlation) could provide additional insight into the relationship between DM and disease severity. Given the complexity of the dataset— multiple cultivars, harvest dates, storage periods, and disease types—we opted to focus on comparative analyses and modeling approaches that allowed us to directly assess these factors and their interactions. Our primary objective was to evaluate the combined effects of variety, harvest timing, and storage duration on postharvest disease development across multiple seasons. Additionally, the relationship between DM and disease severity appeared to vary across cultivars, seasons, and disease types, making it difficult to capture these patterns with a single global correlation analysis. As mentioned, in the current study, we chose to emphasize an integrative interpretation of these relationships and incorporate them into the overall discussion of the results. We appreciate this valuable suggestion and agree that targeted correlation analyses within specific cultivar–season combinations are an interesting direction for future work.

**Comment 2:** I suggest replacing in the title ‘disorders’ with ‘vascular streaking’, since only this physiological disorder is addressed in this study.

**Response 2:** We accepted the reviewers' suggestions and decided to replace ‘disorder’ with vascular streaking in the title. We also introduced changes suggested by another reviewer (page 1, lines 1-2).

**Comment 3.** L151. I think it would be highly illustrative to provide for each variety and cold storage period (1, 3, and 6 weeks), the number of days at 20 ºC and 90 % RH that the fruit needed to get ripe after being removed from storage at 5 ºC and 90 % RH. In other words, the time at 20 ºC between cold storage and disease severity assessment.

**Response 3**: We acknowledge the reviewer’s suggestion; however, the information regarding the number of days required for fruit to ripen at 20 °C following removal from cold storage is not included in this manuscript. These data are part of a separate study that specifically examines the interaction between cold storage duration, avocado variety, and postharvest physiological responses—including ripening behavior—under the same experimental conditions. For this reason, we have intentionally reserved this information for that dedicated publication, where it is presented within a comprehensive postharvest physiology framework.

**Comment 4:** L269. Replace ‘disinfested’ with ‘disinfected’.

**Response 4:** This correction was performed through the text (lines 324 and 327)

**Comment 5:** L568-569. Add the corresponding reference number to literature citations.

**Response 5:** Thank you for detecting this issue. The reference number was incorporated into the manuscript along with the relevant literature citation.
