# Supplementary material for: Influence of fruit maturity and variety on seasonal postharvest fungal diseases and vascular streaking in California Avocados
Source: PLoS One. 2026 Jun 25;21(6):e0351752. doi: 10.1371/journal.pone.0351752 (PMC13298749; doi:10.1371/journal.pone.0351752)
Supplement: Peer review history

# Peer review: Decision Letter 0

**DOI:** 10.1371/journal.pone.0351752.r001

**Authors:** Raed Haleem

5 Apr 2026

PONE-D-26-05513

Seasonal Influence of Fruit Maturity and Variety on Postharvest Fungal Diseases and Disorders of Avocados in California

PLOS One

Dear Dr. Camiletti,

Thank you for submitting your manuscript to PLOS ONE. After careful consideration, we feel that it has merit but does not fully meet PLOS ONE’s publication criteria as it currently stands. Therefore, we invite you to submit a revised version of the manuscript that addresses the points raised during the review process.

Please submit your revised manuscript by May 20 2026 11:59PM. If you will need more time than this to complete your revisions, please reply to this message or contact the journal office at plosone@plos.org. When you're ready to submit your revision, log on to https://www.editorialmanager.com/pone/ and select the 'Submissions Needing Revision' folder to locate your manuscript file.

Please include the following items when submitting your revised manuscript:

- A letter that responds to each point raised by the academic editor and reviewer(s). You should upload this letter as a separate file labeled 'Response to Reviewers'.
- A marked-up copy of your manuscript that highlights changes made to the original version. You should upload this as a separate file labeled 'Revised Manuscript with Track Changes'.
- An unmarked version of your revised paper without tracked changes. You should upload this as a separate file labeled 'Manuscript'.

If you would like to make changes to your financial disclosure, please include your updated statement in your cover letter. Guidelines for resubmitting your figure files are available below the reviewer comments at the end of this letter.

If applicable, we recommend that you deposit your laboratory protocols in protocols.io to enhance the reproducibility of your results. Protocols.io assigns your protocol its own identifier (DOI) so that it can be cited independently in the future. For instructions see: https://journals.plos.org/plosone/s/submission-guidelines#loc-laboratory-protocols. Additionally, PLOS ONE offers an option for publishing peer-reviewed Lab Protocol articles, which describe protocols hosted on protocols.io. Read more information on sharing protocols at https://plos.org/protocols?utm_medium=editorial-email&utm_source=authorletters&utm_campaign=protocols.

As the corresponding author, your ORCID iD is verified in the submission system and will appear in the published article. PLOS supports the use of ORCID, and we encourage all coauthors to register for an ORCID iD and use it as well. Please encourage your coauthors to verify their ORCID iD within the submission system before final acceptance, as unverified ORCID iDs will not appear in the published article. *Only*  the individual author can complete the verification step; PLOS staff *cannot*  verify ORCID iDs on behalf of authors.

We look forward to receiving your revised manuscript.

Kind regards,

Raed Abduljabbar Haleem, Ph.D

Academic Editor

PLOS One

**Journal Requirements:**

When submitting your revision, we need you to address these additional requirements.

1. Please ensure that your manuscript meets PLOS ONE's style requirements, including those for file naming. The PLOS ONE style templates can be found at

https://journals.plos.org/plosone/s/file?id=wjVg/PLOSOne_formatting_sample_main_body.pdf and

https://journals.plos.org/plosone/s/file?id=ba62/PLOSOne_formatting_sample_title_authors_affiliations.pdf

2. Please note that PLOS One has specific guidelines on code sharing for submissions in which author-generated code underpins the findings in the manuscript. In these cases, we expect all author-generated code to be made available without restrictions upon publication of the work. Please review our guidelines at https://journals.plos.org/plosone/s/materials-and-software-sharing#loc-sharing-code and ensure that your code is shared in a way that follows best practice and facilitates reproducibility and reuse.

3. Thank you for stating the following financial disclosure:

“Eurosemillas, S.A. (awarded to M.L.A.).”

Please state what role the funders took in the study.  If the funders had no role, please state: "The funders had no role in study design, data collection and analysis, decision to publish, or preparation of the manuscript."

If this statement is not correct you must amend it as needed.

Please include this amended Role of Funder statement in your cover letter; we will change the online submission form on your behalf.

4. Please note that funding information should not appear in any section or other areas of your manuscript. We will only publish funding information present in the Funding Statement section of the online submission form. Please remove any funding-related text from the manuscript.

5. In the online submission form you indicate that your data is not available for proprietary reasons and have provided a contact point for accessing this data. Please note that your current contact point is a co-author on this manuscript. According to our Data Policy, the contact point must not be an author on the manuscript and must be an institutional contact, ideally not an individual. Please revise your data statement to a non-author institutional point of contact, such as a data access or ethics committee, and send this to us via return email. Please also include contact information for the third party organization, and please include the full citation of where the data can be found.

6. Please include your full ethics statement in the ‘Methods’ section of your manuscript file. In your statement, please include the full name of the IRB or ethics committee who approved or waived your study, as well as whether or not you obtained informed written or verbal consent. If consent was waived for your study, please include this information in your statement as well.

7. We note that you have referenced (DM pattern observed in our study and previous unpublished data.) which has currently not yet been accepted for publication. Please remove this from your References and amend this to state in the body of your manuscript: (ie “Bewick et al. [Unpublished]”) as detailed online in our guide for authors

http://journals.plos.org/plosone/s/submission-guidelines#loc-reference-style

8. We note that Supporting Figures 1, 2, and 3 in your submission contain copyrighted images. All PLOS content is published under the Creative Commons Attribution License (CC BY 4.0), which means that the manuscript, images, and Supporting Information files will be freely available online, and any third party is permitted to access, download, copy, distribute, and use these materials in any way, even commercially, with proper attribution. For more information, see our copyright guidelines: http://journals.plos.org/plosone/s/licenses-and-copyright.

We require you to either (1) present written permission from the copyright holder to publish these figures specifically under the CC BY 4.0 license, or (2) remove the figures from your submission:

1. You may seek permission from the original copyright holder of Supporting Figures 1, 2, and 3  to publish the content specifically under the CC BY 4.0 license.

We recommend that you contact the original copyright holder with the Content Permission Form (http://journals.plos.org/plosone/s/file?id=7c09/content-permission-form.pdf) and the following text:

“I request permission for the open-access journal PLOS ONE to publish XXX under the Creative Commons Attribution License (CCAL) CC BY 4.0 (http://creativecommons.org/licenses/by/4.0/). Please be aware that this license allows unrestricted use and distribution, even commercially, by third parties. Please reply and provide explicit written permission to publish XXX under a CC BY license and complete the attached form.”

Please upload the completed Content Permission Form or other proof of granted permissions as an "Other" file with your submission.

In the figure caption of the copyrighted figure, please include the following text: “Reprinted from [ref] under a CC BY license, with permission from [name of publisher], original copyright [original copyright year].”

2. If you are unable to obtain permission from the original copyright holder to publish these figures under the CC BY 4.0 license or if the copyright holder’s requirements are incompatible with the CC BY 4.0 license, please either i) remove the figure or ii) supply a replacement figure that complies with the CC BY 4.0 license. Please check copyright information on all replacement figures and update the figure caption with source information. If applicable, please specify in the figure caption text when a figure is similar but not identical to the original image and is therefore for illustrative purposes only.

9. If the reviewer comments include a recommendation to cite specific previously published works, please review and evaluate these publications to determine whether they are relevant and should be cited. There is no requirement to cite these works unless the editor has indicated otherwise.

10. Please review your reference list to ensure that it is complete and correct. If you have cited papers that have been retracted, please include the rationale for doing so in the manuscript text, or remove these references and replace them with relevant current references. Any changes to the reference list should be mentioned in the rebuttal letter that accompanies your revised manuscript. If you need to cite a retracted article, indicate the article’s retracted status in the References list and also include a citation and full reference for the retraction notice.

[Note: HTML markup is below. Please do not edit.]

Reviewers' comments:

Reviewer's Responses to Questions

**Comments to the Author**

1. Is the manuscript technically sound, and do the data support the conclusions?

The manuscript must describe a technically sound piece of scientific research with data that supports the conclusions. Experiments must have been conducted rigorously, with appropriate controls, replication, and sample sizes. The conclusions must be drawn appropriately based on the data presented.

Reviewer #1: Yes

Reviewer #2: Yes

Reviewer #3: Yes

**********

2. Has the statistical analysis been performed appropriately and rigorously?

Reviewer #1: Yes

Reviewer #2: Yes

Reviewer #3: Yes

**********

3. Have the authors made all data underlying the findings in their manuscript fully available?

The PLOS Data policy requires authors to make all data underlying the findings described in their manuscript fully available without restriction, with rare exception (please refer to the Data Availability Statement in the manuscript PDF file). The data should be provided as part of the manuscript or its supporting information, or deposited to a public repository. For example, in addition to summary statistics, the data points behind means, medians and variance measures should be available. If there are restrictions on publicly sharing data—e.g. participant privacy or use of data from a third party—those must be specified.

Reviewer #1: Yes

Reviewer #2: Yes

Reviewer #3: Yes

**********

4. Is the manuscript presented in an intelligible fashion and written in standard English?

PLOS ONE does not copyedit accepted manuscripts, so the language in submitted articles must be clear, correct, and unambiguous. Any typographical or grammatical errors should be corrected at revision, so please note any specific errors here.

Reviewer #1: Yes

Reviewer #2: Yes

Reviewer #3: Yes

**********

5. Review Comments to the Author

Please use the space provided to explain your answers to the questions above. You may also include additional comments for the author, including concerns about dual publication, research ethics, or publication ethics. (Please upload your review as an attachment if it exceeds 20,000 characters)

Reviewer #1: The research work is mainly descriptive, evaluating the responses of different genetic backgrounds to three main avocado postharvest diseases. The experimental design, methodological approach, and implementation are sound, and the presentation of the results is clear. Further elaboration of the discussion part is suggested.

There is presentation of a considerable results’ dataset, the analysis of which shapes indicative cultivar profiles regarding harvest time and windows, main disease resistance performance, and in relation to DM which function as (the) fruit quality indicator.

Though there are scarce references to molecules (persin) or genetic background (Haas or non-Haas -related) factors that could reason, connect or support phenotypic responses, there is no further research (molecular/genomics) on these factors at the present time.

On the contrary, the molecular (phylogenetic) characterization of the fungal pathogen isolates via multilocus analysis using selected markers per pathogen goes in depth and covers the needs even for “tougher” species complexes like Colletotrichum spp (use of the most informative markers).

It would be useful to report on the fertilization (soil nutrient analyses if available as well) and plant protection regimes followed during the three different seasons. Was there a horizontal regime applied to all cultivars per season or adapted to each cultivar’s specific needs? Was this (either the former or later case) adapted to each season with regards to the climatic conditions of each season? Please evaluate if it would be informative to comment on and also discuss these aspects (fertilization/plant protection followed) with regards to cultivars’ responses to the pathogens (e.g. if there is relevant data available from previous research, or even if these were parameters that you would like to study in the near future).

What I am missing is a conclusive (or suggestion-type) graph or table gathering the information of individual results (DM, Severity scores, putatively Lesion severities as well...) presented in the individual figures and tables. Since these datasets/results are not fed yet in a predictive (DSS-type) model along with other geoclimatic and field-management actions to suggest harvest time(s)/windows, it would probably be interesting to present a preliminary graph or table using e.g. a selected DM value(s) filter and present collectively harvest windows, pathogen/disease risk, responses, etc. for each cultivar. Please evaluate if this could embrace all your results in a collective format that could further lighten your work and based on the graph/table give you the opportunity to discuss and suggest a putative roadmap for cultivars’ best use.

Other specific points to further elaborate/address are the following:

1. For the susceptibility tests there is selection of certain species. Please comment on the basis of your choice (e.g. most prevalent/most prevalent in general/bibliography based, etc.) to support the choice of certain and not all different species identified

2. For susceptibility tests only one isolate (lines 271-272) per species is used. Please evaluate/comment on the probability that cultivar responses may vary in case a second or third isolate from the same species is used.

3. Line 217; please check if reference of Table 1 is correct.

4. Line 407; typo “XXXXX”

5. Lines 466-467; typo single/plural

6. Line 560; does this (most frequent pathogens) refer to the diseases “respectively” or is mentioned in a generalized way?

7. Line 599-600; Does “fruit maturity” refer to DM-related concept or in a generalized way?

8. In Figure 5 “Others” is a notable share of the Relevant Frequency. Could you report (in the manuscript) the most prevalent genera? Please evaluate if this can be also connected to your comment for endophytes (Lines 520-522), and elaborate/discuss if you think it is relevant.

Reviewer #2: This manuscript is good for learning about avocado varieties and their yields, as well as identifying the fungal diseases that affect them. The research is also good because a large number of fungi have been identified.

Reviewer #3: The manuscript reports studies on the etiology of postharvest fruit rots of avocado in California and the influence of avocado cultivar (some commercial cultivars and breeding selections), fruit maturity (as % of dry matter, DM), and cold storage period on disease severity (score 0-3 for stem-end rots and 0-5 for body rots) on fruits harvested monthly for 3 consecutive seasons. The influence on vascular streaking was also addressed. Susceptibility tests were also conducted with fruits artificially inoculated with three selected pathogenic fungal species.

In my opinion, this research is of very high quality. The subject is of great interest, the volume of work is considerable, and the information is new and valuable because gives some keys to the avocado production sector to maximize profit by minimizing postharvest disease incidence. The manuscript is very well written, clear and well organized in subsections. In general, the experiments are well designed and repeated sufficiently, proper statistical analyses were performed, the results are adequately presented in tables and figures, and the data support the conclusions of the authors. The introduction and discussion are complete, and the number and quality of the literature references are appropriate. I am only wondering why a correlation analysis (Spearman??) between DM and disease severity was not performed for each cultivar, harvest date, and perhaps the first storage period. It seems, reading the abstract (L28), that such analysis could be expected as an outcome from the study.

Since I am not familiar with work at the molecular level, I cannot comment on this part of the research, and I recommend the editor to invite additional expert reviewers to address the molecular identification of fungal species and phylogenetic analyses.

Other particular comments are very minor in nature:

• I suggest replacing in the title ‘disorders’ with ‘vascular streaking’, since only this physiological disorder is addressed in this study.

• L151. I think it would be highly illustrative to provide for each variety and cold storage period (1, 3, and 6 weeks), the number of days at 20 ºC and 90 % RH that the fruit needed to get ripe after being removed from storage at 5 ºC and 90 % RH. In other words, the time at 20 ºC between cold storage and disease severity assessment.

• L269. Replace ‘disinfested’ with ‘disinfected’.

• L568-569. Add the corresponding reference number to literature citations.

**********

6. PLOS authors have the option to publish the peer review history of their article (what does this mean?). If published, this will include your full peer review and any attached files.

If you choose “no”, your identity will remain anonymous but your review may still be made public.

**Do you want your identity to be public for this peer review?**  For information about this choice, including consent withdrawal, please see our Privacy Policy.

Reviewer #1: No

Reviewer #2: No

Reviewer #3: **Yes:** Lluís Palou

**********

[NOTE: If reviewer comments were submitted as an attachment file, they will be attached to this email and accessible via the submission site. Please log into your account, locate the manuscript record, and check for the action link "View Attachments". If this link does not appear, there are no attachment files.]

To ensure your figures meet our technical requirements, please review our figure guidelines: https://journals.plos.org/plosone/s/figures

You may also use PLOS’s free figure tool, NAAS, to help you prepare publication quality figures: https://journals.plos.org/plosone/s/figures#loc-tools-for-figure-preparation.

NAAS will assess whether your figures meet our technical requirements by comparing each figure against our figure specifications.

Attachment Submitted filename: Manuscript_avocado_seasonal_PLOS.docx

# Author comment: Author response to Decision Letter 1

**DOI:** 10.1371/journal.pone.0351752.r002

22 May 2026

Response to Editorial comments

Dear Editor,

Please find the detailed responses below and the corresponding revisions and corrections in the re-submitted files.

Comment 1: Please ensure that your manuscript meets PLOS ONE's style requirements, including those for file naming.

Response 1: The manuscript body, title, authors, and affiliation formats were checked. The current address for author C. M. Asensio was added as follows (Page 1, lines 14-15):

“# a Department of Food Science and Human Nutrition, University of Illinois Urbana-Champaign, Urbana, IL 618101, U.S.A.”

Comment 2: Please note that PLOS One has specific guidelines on code sharing for submissions in which author-generated code underpins the findings in the manuscript. In these cases, we expect all author-generated code to be made available without restrictions upon publication of the work.

Response 2: According to the journal guidelines, a repository for the codes used in this study was generated. The code used to generate the results in this study is publicly available at Zenodo: https://zenodo.org/records/20218289 .

Comment 3: Thank you for stating the following financial disclosure: “Eurosemillas, S.A. (awarded to M.L.A.).” Please state what role the funders took in the study. If the funders had no role, please state: "The funders had no role in study design, data collection and analysis, decision to publish, or preparation of the manuscript."

Response 3: Additional information was incorporated into the financial disclosure as suggested. “Funding: Eurosemillas, S.A. (awarded to M.L.A.). The funders had no role in study design, data collection and analysis, decision to publish, or preparation of the manuscript.”

Comment 4. Please note that funding information should not appear in any section or other areas of your manuscript. We will only publish funding information present in the Funding Statement section of the online submission form. Please remove any funding-related text from the manuscript.

Response 4: Thanks, the financial disclosure statement was removed from the manuscript as suggested.

Comment 5: In the online submission form, you indicate that your data is not available for proprietary reasons and have provided a contact point for accessing this data. Please note that your current contact point is a co-author on this manuscript. According to our Data Policy, the contact point must not be an author on the manuscript and must be an institutional contact, ideally not an individual. Please revise your data statement to a non-author institutional point of contact, such as a data access or ethics committee, and send this to us via return email. Please also include contact information for the third party organization, and please include the full citation of where the data can be found.

Response 5: Thank you for your clarifications regarding the data availability requirements. We have deposited the dataset and associated R scripts in the Zenodo repository and obtained a DOI for the record. The repository includes all data and code necessary to reproduce the analyses reported in the manuscript. At this stage, the files are under a temporary embargo during the peer-review process. However, we confirm that all data and code will be made fully publicly available without restriction upon publication, in full compliance with PLOS ONE’s Data Availability policy.

The following information was included in our Data Availability Statement:

“DNA sequence data for all fungal isolates analyzed in this study have been deposited in GenBank, and accession numbers are provided in the manuscript and Supporting Information. We are currently sharing data specific to our analyses so that they can be legally distributed. All data and code supporting the findings will be publicly available upon publication of the manuscript at Zenodo: https://zenodo.org/records/20313537.

Comment 6: Please include your full ethics statement in the ‘Methods’ section of your manuscript file. In your statement, please include the full name of the IRB or ethics committee who approved or waived your study, as well as whether or not you obtained informed written or verbal consent. If consent was waived for your study, please include this information in your statement as well.

Comment 6: Thank you for your comment. We would like to clarify that this study did not involve human participants or vertebrate animals. The research was conducted on plant materials (avocado) and associated pathogens. Therefore, Institutional Review Board (IRB) approval and informed consent are not applicable to this research.

The following information is included in the Ethics Statement, because the study involved “Field Research”:

“Field sampling of avocado fruit was conducted in a University of California–managed research station in California. Permission to collect fruit and plant material was granted by the University of California, Riverside Agricultural Operations. No government permits were required for this work.”

Comment 7: We note that you have referenced (DM pattern observed in our study and previous unpublished data.) which has currently not yet been accepted for publication. Please remove this from your References and amend this to state in the body of your manuscript: (ie “Bewick et al. [Unpublished]”) as detailed online in our guide for authors

Response 7: Thank you for your comment. We have modified this to meet the journal guidelines. Unpublished data were cited in the manuscript as “Camiletti et al. [Unpublished]” (page 25, lines 485-486).

Comment 8: We note that Supporting Figures 1, 2, and 3 in your submission contain copyrighted images. All PLOS content is published under the Creative Commons Attribution License (CC BY 4.0), which means that the manuscript, images, and Supporting Information files will be freely available online, and any third party is permitted to access, download, copy, distribute, and use these materials in any way, even commercially, with proper attribution. For more information, see our copyright guidelines: http://journals.plos.org/plosone/s/licenses-and-copyright.

We require you to either (1) present written permission from the copyright holder to publish these figures specifically under the CC BY 4.0 license, or (2) remove the figures from your submission:

Response 8: Thank you for your comment regarding Supporting Figures 1–3. We would like to clarify that the figures in question are original and author-generated, and the copyright is held by the first author of the manuscript. These images have not been reproduced from any previously published source and do not include third-party copyrighted material. As such, no additional copyright permission is required. We confirm that these figures can be published under the Creative Commons Attribution (CC BY 4.0) license in accordance with PLOS ONE’s policies. Please let us know if any further clarification is needed.

Comment 9: If the reviewer comments include a recommendation to cite specific previously published works, please review and evaluate these publications to determine whether they are relevant and should be cited. There is no requirement to cite these works unless the editor has indicated otherwise.

Response 9: We appreciate the clarification. We were suggested to include a citation in our literature. That change is reflected in the manuscript

Comment 10: Please review your reference list to ensure that it is complete and correct. If you have cited papers that have been retracted, please include the rationale for doing so in the manuscript text, or remove these references and replace them with relevant current references. Any changes to the reference list should be mentioned in the rebuttal letter that accompanies your revised manuscript. If you need to cite a retracted article, indicate the article’s retracted status in the References list and also include a citation and full reference for the retraction notice.

Response 10: Thank you for your comment. /

Response to Review #1

Dear reviewer,

Thank you for your insightful comments. Please find the detailed responses below and the corresponding revisions and corrections in the re-submitted files.

Comment 1: The research work is mainly descriptive, evaluating the responses of different genetic backgrounds to three main avocado postharvest diseases. The experimental design, methodological approach, and implementation are sound, and the presentation of the results is clear. Further elaboration of the discussion part is suggested.

Response 1: We appreciate the positive feedback and revised the Discussion to further elaborate on key findings and study limitations (lines 631-635; 658-660; 665-668; 671-673).

Comment 2: There is presentation of a considerable results’ dataset, the analysis of which shapes indicative cultivar profiles regarding harvest time and windows, main disease resistance performance, and in relation to DM which function as (the) fruit quality indicator

Response 2: We thank the reviewer for recognizing the value of the dataset and the relevance of the cultivar-specific analyses linking harvest timing, disease response, and dry matter accumulation.

Comment 3: Though there are scarce references to molecules (persin) or genetic background (Haas or non- Haas -related) factors that could reason, connect or support phenotypic responses, there is no further research (molecular/genomics) on these factors at the present time.

Response 3: We appreciate this insightful observation and agree that further molecular and genomic studies would be valuable to better explain the phenotypic responses observed.

Comment 4: On the contrary, the molecular (phylogenetic) characterization of the fungal pathogen isolates via multilocus analysis using selected markers per pathogen goes in depth and covers the needs even for “tougher” species complexes like Colletotrichum spp (use of the most informative markers).

Response 4: We thank the reviewer for acknowledging the depth of the multilocus phylogenetic analyses and the use of informative molecular markers for pathogen characterization.

Comment 5: It would be useful to report on the fertilization (soil nutrient analyses if available as well) and plant protection regimes followed during the three different seasons. Was there a horizontal regime applied to all cultivars per season or adapted to each cultivar’s specific needs? Was this (either the former or later case) adapted to each season with regards to the climatic conditions of each season? Please evaluate if it would be informative to comment on and also discuss these aspects (fertilization/plant protection followed) with regards to cultivars’ responses to the pathogens (e.g. if there is relevant data available from previous research, or even if these were parameters that you would like to study in the near future).

Response 5: We thank the reviewer for this valuable suggestion. We added information on fertilization and plant protection practices to the Materials and Methods section (lines 161-174) and briefly expanded the Discussion (lines 665-668) to clarify that all cultivars were maintained under the same standard minimum fertilization program, with no fungicide or insecticide applications.

Comment 6: What I am missing is a conclusive (or suggestion-type) graph or table gathering the information of individual results (DM, Severity scores, putatively Lesion severities as well...) presented in the individual figures and tables. Since these datasets/results are not fed yet in a predictive (DSStype) model along with other geoclimatic and field-management actions to suggest harvest time(s)/windows, it would probably be interesting to present a preliminary graph or table using e.g. a selected DM value(s) filter and present collectively harvest windows, pathogen/disease risk, responses, etc. for each cultivar. Please evaluate if this could embrace all your results in a collective format that could further lighten your work and based on the graph/table give you the opportunity to discuss and suggest a putative roadmap for cultivars’ best use.

Response 6: We thank the reviewer for this helpful suggestion. We added a new summary table (Table 5) integrating seasonal dry matter accumulation patterns, observed disease trends, and preliminary harvest considerations for each cultivar and selection (lines 726-728).

Comment 7: 1. For the susceptibility tests there is selection of certain species. Please comment on the basis of your choice (e.g. most prevalent/most prevalent in general/bibliography based, etc.) to support the choice of certain and not all different species identified.

Response 7: We thank the reviewer for this comment. We added clarification to the Materials and Methods (lines 331-332) indicating that the pathogens selected for susceptibility assays corresponded to the most prevalent species identified during the surveys.

Comment 8: For susceptibility tests only one isolate (lines 271-272) per species is used. Please evaluate/comment on the probability that cultivar responses may vary in case a second or third isolate from the same species is used.

Response 8: We thank the reviewer for this important observation. We added a sentence to the Discussion acknowledging this limitation and noting that future studies with multiple isolates per species would help evaluate potential intraspecific variability in aggressiveness and cultivar responses (lines 671-673).

Comment 9: Line 217; please check if reference of Table 1 is correct.

Response 9: The incorrect table reference was removed.

Comment 10: 4. Line 407; typo “XXXXX”

Response 10: We thank the reviewer for noting this. The placeholder was corrected and the missing information was added (line 496).

Comment 11: Lines 466-467; typo single/plural

Response 11: We thank the reviewer for noting this. The sentence was corrected (line 568).

Comment 12: Line 560; does this (most frequent pathogens) refer to the diseases “respectively” or is mentioned in a generalized way?

Response 12: This was intended in a general sense, as pathogen predominance can vary by disease.

Comment 13: Line 599-600; Does “fruit maturity” refer to DM-related concept or in a generalized way?

Response 13: We revised the sentence to clarify that this refers to DM-based maturity (lines 721-722).

Comment 14: In Figure 5 “Others” is a notable share of the Relevant Frequency. Could you report (in the manuscript) the most prevalent genera? Please evaluate if this can be also connected to your comment for endophytes (Lines 520-522), and elaborate/discuss if you think it is relevant.

Response 14: We thank the reviewer for this valuable suggestion. We clarified the main genera included in “Others” and added a sentence to the Discussion (lines 631-635) noting that some of these fungi may occur as opportunistic endophytes or secondary colonizers, although their specific role in postharvest disease development was beyond the scope of this study.

Response to Review #2

Dear reviewer,

Thank you for your insightful comments. Please find the detailed responses below and the corresponding revisions and corrections in the re-submitted files. We accepted most of the minor comments throughout the text; you can see them in the track-changed version.

General Comment: This manuscript is good for learning about avocado varieties and their yields, as well as identifying the fungal diseases that affect them. The research is also good because a large number of fungi have been identified.

Response: We thank you for the very positive and thoughtful comment.

Comment 1: Suggested to change title to “Fruit Maturity and Variety's Seasonal Effects on Avocado Postharvest Fungal Diseases and Disorders in California”.

Response 1: We thank the reviewer for the suggestion. We modified the current title according to your and other reviewers' suggestions.

Comment 2: In the abstract “You must mention the aims of study , the variety of avocados , mention the primers and then the results”.

Response 2: We thank the reviewer for this suggestion. The first part of the abstract was revised to include the study aims, avocado varieties, molecular regions used, and main results (lines 23-28).

Comment 3: Introduction , reviewer suggested d

Attachment Submitted filename: Response to reviewers R1 final.docx

# Editor report: Decision Letter 1

**DOI:** 10.1371/journal.pone.0351752.r003

**Authors:** Raed Haleem, Raed Haleem

2 Jun 2026

Influence of Fruit Maturity and Variety on Seasonal Postharvest Fungal Diseases and Vascular Streaking in California Avocados

PONE-D-26-05513R1

Dear Dr. Camiletti,

We’re pleased to inform you that your manuscript has been judged scientifically suitable for publication and will be formally accepted for publication once it meets all outstanding technical requirements.

Within one week, you’ll receive an e-mail detailing the required amendments. When these have been addressed, you’ll receive a formal acceptance letter and your manuscript will be scheduled for publication.

An invoice will be generated when your article is formally accepted. Please note, if your institution has a publishing partnership with PLOS and your article meets the relevant criteria, all or part of your publication costs will be covered. Please make sure your user information is up-to-date by logging into Editorial Manager at Editorial Manager® and clicking the ‘Update My Information' link at the top of the page. For questions related to billing, please contact billing support.

If your institution or institutions have a press office, please notify them about your upcoming paper to help maximize its impact. If they’ll be preparing press materials, please inform our press team as soon as possible -- no later than 48 hours after receiving the formal acceptance. Your manuscript will remain under strict press embargo until 2 pm Eastern Time on the date of publication. For more information, please contact onepress@plos.org.

Kind regards,

Raed Abduljabbar Haleem, Ph.D

Academic Editor

PLOS One

Additional Editor Comments (optional):

Reviewers' comments:

# Editor report: Acceptance letter

**DOI:** 10.1371/journal.pone.0351752.r004

**Authors:** Raed Haleem, Raed Haleem

PONE-D-26-05513R1

PLOS One

Dear Dr. Camiletti,

I'm pleased to inform you that your manuscript has been deemed suitable for publication in PLOS One. Congratulations! Your manuscript is now being handed over to our production team.

At this stage, our production department will prepare your paper for publication. This includes ensuring the following:

* All references, tables, and figures are properly cited

* All relevant supporting information is included in the manuscript submission,

* There are no issues that prevent the paper from being properly typeset

You will receive further instructions from the production team, including instructions on how to review your proof when it is ready. Please keep in mind that we are working through a large volume of accepted articles, so please give us a few days to review your paper and let you know the next and final steps.

Lastly, if your institution or institutions have a press office, please let them know about your upcoming paper now to help maximize its impact. If they'll be preparing press materials, please inform our press team within the next 48 hours. Your manuscript will remain under strict press embargo until 2 pm Eastern Time on the date of publication. For more information, please contact onepress@plos.org.

You will receive an invoice from PLOS for your publication fee after your manuscript has reached the completed accept phase. If you receive an email requesting payment before acceptance or for any other service, this may be a phishing scheme. Learn how to identify phishing emails and protect your accounts at https://explore.plos.org/phishing.

If we can help with anything else, please email us at customercare@plos.org.

Thank you for submitting your work to PLOS ONE and supporting open access.

Kind regards,

PLOS ONE Editorial Office Staff

on behalf of

Dr. Raed Abduljabbar Haleem

Academic Editor

PLOS One
